# Ultra-fast triplet-triplet-annihilation-mediated high-lying reverse intersystem crossing triggered by participation of nπ*-featured excited states

**DOI:** 10.1038/s41467-022-34573-2

**Published:** 2022-11-12

**Authors:** Yanju Luo, Kai Zhang, Zhenming Ding, Ping Chen, Xiaomei Peng, Yihuan Zhao, Kuan Chen, Chuan Li, Xujun Zheng, Yan Huang, Xuemei Pu, Yu Liu, Shi-Jian Su, Xiandeng Hou, Zhiyun Lu

**Affiliations:** 1 Key Laboratory of Green Chemistry and Technology (Ministry of Education), College of Chemistry, Sichuan University, Chengdu, 610064 P. R. China; 2 Analytical & Testing Centre, Sichuan University, Chengdu, 610064 P. R. China; 3 Jiangsu Engineering Laboratory of Light-Electricity-Heat Energy-Converting Materials and Applications, Changzhou University, Changzhou, 213164 P. R. China; 4 Chongqing key Laboratory of Micro&Nano Structure Optoelectronics, School of Physical Science and Technology, Southwest University, Chongqing, 400715 P. R. China; 5 State Key Laboratory of Luminescent Materials and Devices and Institute of Polymer Optoelectronic Materials and Devices, South China University of Technology, Guangzhou, 510640 P. R. China

**Keywords:** Electronic devices, Optical materials

## Abstract

The harvesting of ‘hot’ triplet excitons through high-lying reverse intersystem crossing mechanism has emerged as a hot research issue in the field of organic light-emitting diodes. However, if high-lying reverse intersystem crossing materials lack the capability to convert ‘cold’ T_1_ excitons into singlet ones, the actual maximum exciton utilization efficiency would generally deviate from 100%. Herein, through comparative studies on two naphthalimide-based compounds CzNI and TPANI, we revealed that the ‘cold’ T_1_ excitons in high-lying reverse intersystem crossing materials can be utilized effectively through the triplet-triplet annihilation-*mediated* high-lying reverse intersystem crossing process if they possess certain triplet-triplet upconversion capability. Especially, quite effective triplet-triplet annihilation-*mediated* high-lying reverse intersystem crossing can be triggered by endowing the high-lying reverse intersystem crossing process with a ^3^ππ*→^1^nπ* character. By taking advantage of the permanent orthogonal orbital transition effect of ^3^ππ*→^1^nπ*, spin–orbit coupling matrix elements of ca. 10 cm^−1^ can be acquired, and hence ultra-fast mediated high-lying reverse intersystem crossing process with rate constant over 10^9^ s^−1^ can be realized.

## Abstract (web summary)

Here, the authors investigate naphthalimide-based compounds with cold triplet excitons utilized by triplet-triplet annihilation-mediated high-lying reverse intersystem crossing, and realize rate constant of over 10^9^ s^−1^ with exciton utilization efficiency of 46.7% in organic light-emitting diodes.

## Introduction

Owing to its integrated high triplet exciton utilization efficiency, rapid triplet exciton conversion, and fast singlet exciton radiative deactivation, high-lying reverse intersystem crossing (hRISC) mechanism, i.e., RISC process occurring from a high-lying triplet state (T_*n*_, *n* ≥ 2) to a singlet state (S_*m*_, *m* ≥ 1), has attracted much recent attention in the field of organic light-emitting diode (OLED) materials^1–4^. To trigger a rapid and efficient hRISC process, an ultra-small energy splitting ($\Delta {E_{{{{\rm{S}}}}_{m}-{{{{\rm{T}}}}_{n}}}}$) and a relatively large spin–orbit coupling matrix element (SOCME) between the T_*n*_ and S_*m*_ states, and a sufficiently large energy gap between the T_*n*_ and T_1_ states ($\Delta {E_{{{{\rm{T}}}}_{n}-{{{{\rm{T}}}}_{1}}}}$) are indispensible^3,5,6^. On the premise that the internal conversion (IC) process from the T_*n*_ to T_1_ states can be blocked thoroughly, and no T_1_ excitons can be formed through direct electrical injection (i.e., ‘cold’ T_1_ excitons), the theoretical maximum exciton utilization efficiency (EUE_max_) of hRISC-OLED material is as high as 100%^3,7–9^. However, as most hRISC materials suffer from relatively small $\Delta {E_{{{{\rm{T}}}}_{n}-{{{{\rm{T}}}}_{1}}}}$ (< 2 eV^8,10–12^), it is quite difficult to block the IC process of T_*n*_→T_1_ completely. Additionally, there were evidences confirming the presence of ‘cold’ T_1_ excitons in OLEDs^13^. Therefore, once a hRISC material lacks the capability to convert T_1_ excitons into singlet ones, the actual EUE_max_ should deviate from 100%.

**Fig. 1:**
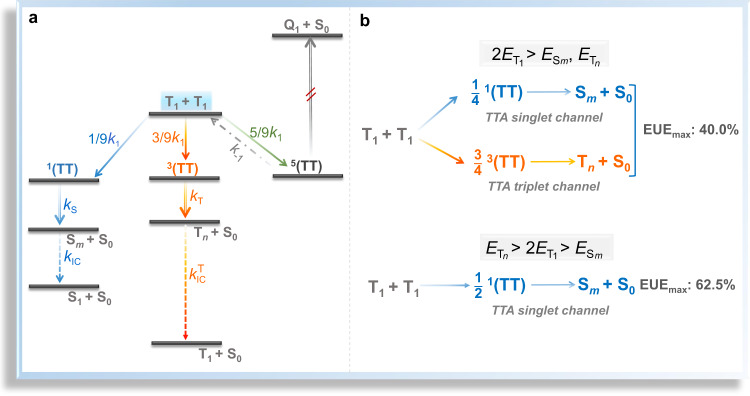
TTA mechanism and EUE_max_ of the general TTA model. **a** Energy-level diagram illustrating the mechanism of TTA. **b** EUE_max_ of the general TTA model with its triplet channel opened or closed, respectively. ^1^(TT), ^3^(TT), and ^5^(TT) are singlet-, triplet-, and quintet-featured intermediate states, respectively; S_1_, T_1_, and S_0_ are the lowest excited singlet state, the lowest excited triplet state and ground state, respectively; S_*m*_ and T_*n*_ are higher-lying singlet and triplet states, respectively; *k*_1_ and *k*_*−*1_ are the rate constants of the generation of TT pair via the collision of two T_1_ excitons and the dissociation of TT pair, respectively; *k*_S_ and *k*_T_ are the rate constants of internal conversion (IC) processes from ^1^(TT) and ^3^(TT) intermediate states to S_*m*_ and T_*n*_, respectively; *k*_IC_ and ${k}_{{{{{{\rm{IC}}}}}}}^{{{{{{\rm{T}}}}}}}$ are the rate constants of IC processes from S_*m*_ to S_1_ and from T_*n*_ to T_1_, respectively.

On the other hand, triplet-triplet annihilation (TTA), a photophysical process that two low-energy T_1_ excitons fuse into one high-energy exciton^14,15^, is another triplet exciton utilization mechanism widely used in OLEDs^16–19^. As illustrated in Fig. 1a, generally, TTA-upconversion (TTU) is expressed with the following two steps: (1) two independent T_1_ excitons collide to form a spin-correlative triplet-triplet pair (TT);^14^ (2) the resultant TT pair can be converted into a high-energy excited state and a ground state^14^. In the first step, since the spin of the TT is identical to the total spins of the initial two separated triplets^14^, the formed TT will consist of nine spin states, namely, one singlet-featured ^1^(TT), three triplet-featured ^3^(TT), and five quintet (Q)-featured ^5^(TT)^14,15,18,20–22^. In the second step, the ^1^(TT), ^3^(TT) and ^5^(TT) can be converted into a high-energy singlet exciton (S_*m*_) (singlet-channel), a high-energy triplet exciton (T_*n*_) (triplet-channel) and a high-energy quintuplet exciton (Q_1_) (quintet channel), respectively, under the premise of $2{E_{{{{\rm{T}}}}_{1}}} \ge {E_{{{{\rm{S}}}}_{m}}}, 2{E_{{{{\rm{T}}}}_{1}}} \ge {E_{{{{\rm{T}}}}_{n}}}$, and $2{E_{{{{\rm{T}}}}_{1}}} \ge {E_{{{{\rm{Q}}}}_{1}}}$ in sequence^14^. Yet for most organic molecules, their Q_1_ energy levels are too high to render a highly efficient TTU quintet channel^15,18,23,24^, thereupon the ^5^(TT) will dissociate apart into two T_1_ excitons, which will be finally converted into ^1^(TT) and ^3^(TT) through collision^14,23,24^. As a consequence, for a TTU process lacking of effective quintet channel, the resulting TT will consist of four spin states, i.e., one ^1^(TT) state and three ^3^(TT) states (depicted in Fig. 1b, top)^14,23,24^. It should be pointed out that although in solid state, TT pair is generally expected to undergo rapid dissociation, recently, there have been some reports on the observation of direct photoluminescence (PL) emission from TT^25,26^, indicating that TT pair may also possess a relatively long lifetime. Nevertheless, whether TT pair can rapidly dissociate or not, through singlet-channel of TTU, two T_1_ excitons can be converted into one S_1_ excitons. Yet for the triplet channel of TTU, since the T_*n*_-to-S_1_ hRISC conversion is generally assumed to be quite inefficient due to its spin-disallowed nature, the final TTA-induced singlet yield in this channel is much lower than that in the singlet-channel (0.2 *vs*. 0.5)^27^, and thereby the EUE_max_ of the corresponding OLED is only 40.0% (25.0% + 75.0% × 0.2)^15,23,27,28^. Therefore, most of the current work is focused on the construction of OLED materials with blocked TTU triplet channels, i.e., the two energy requirements of $2{E_{{{{\rm{T}}}}_{1}}} \ge {E_{{{{\rm{S}}}}_{m}}}$ and $2{E_{{{{\rm{T}}}}_{1}}} \le {E_{{{{\rm{T}}}}_{n}}}$ should be met simultaneously, so that the EUE_max_ of the OLEDs can reach 62.5% (25.0% + 75.0% × 0.5)^23,24,27,28^. This will, however, pose great difficulties for the rational design of TTA-OLED molecules.

Very recently, there were two encouraging reports that even in TTU-OLEDs whose singlet-channel and triplet-channel are both opened, EUE_max_ of >40.0% can be accessible^21,28^. In 2019, Adachi et al. unveiled that in some anthracene derivatives, the ^3^(TT) state produced via the triplet-channel of TTU can be efficiently converted into the S_*m*_ state (T_1_ + T_1_ → ^3^(TT) → S_*m*_ → S_1_) owing to the strong SOC interactions between the ^3^(TT) and the S_*m*_ states, and hence the EUE_max_ can be up to 62.5% in these OLEDs^21^. In 2022, Kim et al. reported their analogous findings that through a cascade process of T_1_ + T_1_ → T_*n*_ → S_*m*_ → S_1_, a TTU-produced T_*n*_ excitons can be transformed into S_*m*_ excitons in an anthracene derivative-based OLED, through which an EUE_max_ of 48.0% has been realized^28^. Therefore, whether the ^3^(TT) state is internally converted into T_*n*_ or not, two T_1_ excitons can be converted into one S_1_ excitons even via the triplet-channel of TTU on the premise that the SOC between ^3^(TT)/T_*n*_ and S_*m*_ is relatively strong, hence the EUE_max_ of OLEDs with opened TTU triplet-channel (i.e., $2{E_{{{{\rm{T}}}}_{1}}} \ge {E_{{{{\rm{S}}}}_{m}}}$ and $2{E_{{{{\rm{T}}}}_{1}}} \ge {E_{{{{\rm{T}}}}_{n}}}$) also can exceed 40.0%. Although both the two reports mainly addressed the potential benefits of this mechanism for TTA-OLED materials, based on the fact that the T_*n*_ → S_*m*_ step in this mechanism is also of hRISC feature, we conjectured if this mechanism can be employed to harvest the ‘cold’ T_1_ excitons that are generally wasted in common hRISC-OLEDs. That is, the ‘hot’ T_*n*_ excitons are utilized through a direct hRISC (*d*-hRISC) process, while the ‘cold’ T_1_ excitons are converted into singlet ones either through singlet-channel TTA, or via TTA-*mediated* hRISC (TTA-*m*-hRISC) in the triplet channel. Encouragingly, very recently, it has been revealed that hRISC materials can also possess TTA-upconversion capability^7^. The fact that hRISC and TTA processes can indeed coexist thus ignited our enthusiasm for developing hRISC-OLED materials with additional TTA-*m*-hRISC mechanism.

Nevertheless, so far the only two relevant reports on TTA-*m*-hRISC materials are both based on anthracene derivatives that bear a nearly orthogonal donor-acceptor (D-A) molecular scaffold^21,28^, so that a certain degree of SOCME can be induced between their ππ*-featured charge-transfer (CT) and local excited (LE) states with different spin multiplicities because of the change in the orbital angular momentum. This strategy, however, will lead to a dilemma: (1) due to the structural relaxation, it is quite difficult to maintain the orthogonal ground-state conformation of a compound in its excited state^29^, which adversely affects the large SOCME; (2) even if an orthogonal D-A conformation is obtained in the excited state, it is necessary to avoid the CT state from becoming the S_1_ state, since its forbidden transition will lead to a small radiative rate constant (*k*_f_)^30,31^.

Herein, through comparative studies on two naphthalimide-based compounds CzNI and TPANI, we revealed that the ‘cold’ T_1_ excitons in a hRISC material can be utilized effectively through the TTA-*m*-hRISC mechanism if it is endowed with TTA capability (TPANI). It is noteworthy that the T_*n*_ → S_*m*_ *m*-hRISC process in TPANI shows a ^3^ππ*→^1^nπ* instead of a ^3^ππ*→^1^ππ* character. By taking advantage of the permanent orthogonal orbital transition effect of ^3^ππ*→^1^nπ*, a SOCME of near 10 cm^−1^ can be acquired, and hence an ultra-fast *m*-hRISC process with a rate constant (*k*_*m*-hRISC_) over 10^9 ^s^−1^ can be realized in TPANI. Moreover, as the S_1_ state of TPANI shows hybridized LE and CT (HLCT) characters, a relatively large *k*_f_ of 10^8 ^s^−1^ is readily acquired. Benefiting from this mechanism, in spite of the relatively low photoluminescence quantum efficiency (PLQY) of TPANI (50.6%), a decent maximum external quantum efficiency (EQE_max_) of 7.8% and EUE_max_ of at least 46.7% are acquired in a TPANI-based OLED. These results not only present a promising method to access hRISC materials, but also greatly expand the diversity of constructive units for high-performance TTA-*m*-hRISC-OLED materials.

## Results

### Molecular design and synthesis

2-(4-(*tert*-butyl)phenyl)−6-(9-(4-(*tert*-butyl)phenyl)−9*H*-carbazol-3-yl)−1*H*-benzo[*de*]isoquinoline-1,3(2*H*)-dione (CzNI) and 2-(4-(*tert*-butyl)phenyl)−6-(4-(diphenylamino)phenyl)−1*H*-benzo[*de*]isoquinoline-1,3(2*H*)-dione (TPANI) bearing a naphthalimide-based A subunit and a carbazole-based or a triphenylamine-based D subunit were designed and synthesized. The synthetic route and detailed synthesis of target compound are provided in Supplementary Fig. 1. The molecular structure of target compound was fully characterized by ^1^H and ^13^C nuclear magnetic resonance (NMR) spectroscopies as well as high-resolution mass spectrometry. Single crystal samples of CzNI and TPANI were obtained by slow evaporation of saturated solution from mixed solvents (dichloromethane and ethanol) under room temperature. CzNI and TPANI were purified through three times recrystallization followed by vacuum sublimation.

### Photophysical and electroluminescent properties

**Fig. 2:**
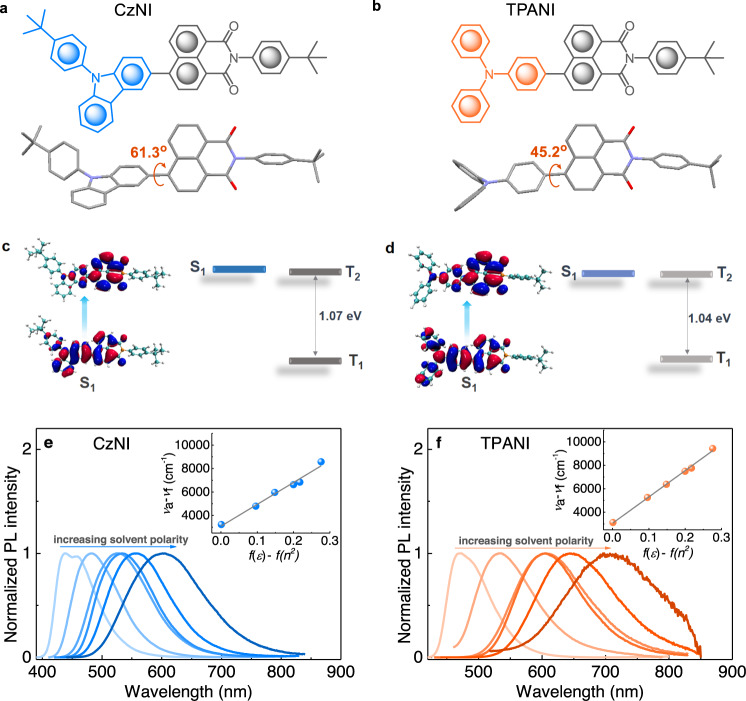
Molecular structures, theoretical calculation results and PL spectra. Molecular structures and single crystal structures of **a** CzNI and **b** TPANI. The natural transition orbital (NTO) pairs and energy levels of **c** CzNI and **d** TPANI for the representative excited states of S_1_, T_1_, and T_2_. Normalized PL spectra of **e** CzNI and **f** TPANI in solvents with different polarity (10^−5^ M, solvents used with increasing polarity are *n*-hexane, butyl ether, ethyl acetate, chloroform, dichloromethane and *N*,*N*-dimethylformamide in sequence). Insets: solvatochromic Lippert–Mataga models of CzNI and TPANI, where *υ*_a_−*υ*_f_ is the Stokes shift, *f*(*ε*)−*f*(*n*^2^) is the orientational polarizability of solvents in which *ε* is the solvent dielectric constant and *n* is the solvent refractive index.

Actually, this ^3^ππ*→^1^nπ* promoted TTA-*m*-hRISC mechanism was discovered accidently when we conducted comparative studies on CzNI and TPANI (as illustrated in Fig. 2). Initially, CzNI and TPANI were designed specifically as normal hRISC-OLED materials that generally possess a HLCT excited state^11,32^. Consistent with our expection, CzNI and TPANI both show a moderately twisted molecular conformation, and their D-A dihedral angles are 61.3° and 45.2°, respectively (Fig. 2a, b), both of which fall within the ideal region required for the formation of HLCT excited state^32,33^. Further density functional theory (DFT) and time-dependent DFT (TD-DFT) calculation results confirmed that the S_1_ states of CzNI and TPANI indeed show typical HLCT transition natures, because their “holes” are delocalized on the D and A units, while the “particles” are mainly distributed on the A moiety (Fig. 2c, d).

The HLCT feature of the S_1_ states of CzNI and TPANI was further confirmed by steady-state and transient photoluminescence (PL) experiments. As depicted in Fig. 2e, f, in nonpolar *n*-hexane, both CzNI and TPANI display a vibrational-structured PL band, indicative of the LE-dominated transition character; with increasing solvent polarity, the two compounds both have their PL spectra red-shifted and widened gradually, together with an obscured fine-structure, manifesting the typical CT-dominated feature of their S_1_ states. Inferred from the fact that the two compounds both show a linear Lippert–Mataga plot (Fig. 2e, f, insets) and a single-exponential PL decay profile with relatively short lifetime (*τ* < 5 ns, vide Supplementary Fig. 5 and Supplementary Table 3), the LE and CT in their S_1_ states should be quasi-equivalently hybridized^5^. Moreover, relatively large *k*_f_s (1.1 × 10^8^ and 1.0 × 10^8^ s^−1^) are realized for CzNI and TPANI owing to their HLCT characters of the S_1_ state.

In addition to the HLCT character of their S_1_ states, both CzNI and TPANI show a relatively large calculated $\Delta {E_{{{{\rm{T}}}}_{2}-{{{{\rm{T}}}}_{1}}}}$ (1.07 and 1.04 eV, vide Fig. 2c, d) and a relatively large experimental $\Delta {E_{{{{\rm{S}}}}_{1}-{{{{\rm{T}}}}_{1}}}}$ (0.74 and 0.56 eV, vide Table 1), making them promising hRISC materials rather than direct RISC materials. However, further electroluminescence (EL) characterization results revealed that a non-doped CzNI-based Device A shows a significantly lower EQE_max_ than the TPANI-based Device B that bears a similar device structure [0.61% *vs*. 2.31%, device structure: ITO/NPB (30 nm)/CBP (3 nm)/emitting layer (EML, 20 nm)/BPhen (50 nm)/LiF (1.2 nm)/Al (120 nm), vide Supplementary Fig. 18], even though the PL quantum yield (QY) of CzNI is slightly higher than that of TPANI in neat film state (61.9% *vs*. 50.6%).

**Table 1:** Photophysical, electrochemical, and thermal stability properties of CzNI and TPANI

Compd.	*τ* [ns]^a^	${E_{{{{\rm{S}}}}_{1}}}/{E_{{{{\rm{T}}}}_{1}}}/\Delta {E_{{{{\rm{S}}}}_{1}-{{{\rm{T}}}_{1}}}}$ [eV]		$\Delta{E_{{{{\rm{T}}}}_{2}-{{{\rm{T}}}_{1}}}}$ [eV]	HOMO/LUMO [eV]^c^	*T*_d_/*T*_m_ [^o^C]
		Exp.^b^	Calcd.	Calcd.		
CzNI	3.69	2.85/2.11/0.74	2.93/1.81/1.12	1.07	‒5.65/‒3.18	418/295
TPANI	4.89	2.63/2.07/0.56	2.77/1.73/1.04	1.04	‒5.39/‒3.18	365/252

^a^Measured in toluene solution under N_2_.

^b^Singlet and triplet energy levels were experimentally estimated from the emission onset of fluorescence spectra in toluene (RT) and the highest energy peak of phosphorescence spectra in iodoethane (77 K), respectively.

^c^HOMO and LUMO energy levels were determined from the onset potential of the oxidation and reduction curves with respect to ferrocene in MeCN.

**Fig. 3:**
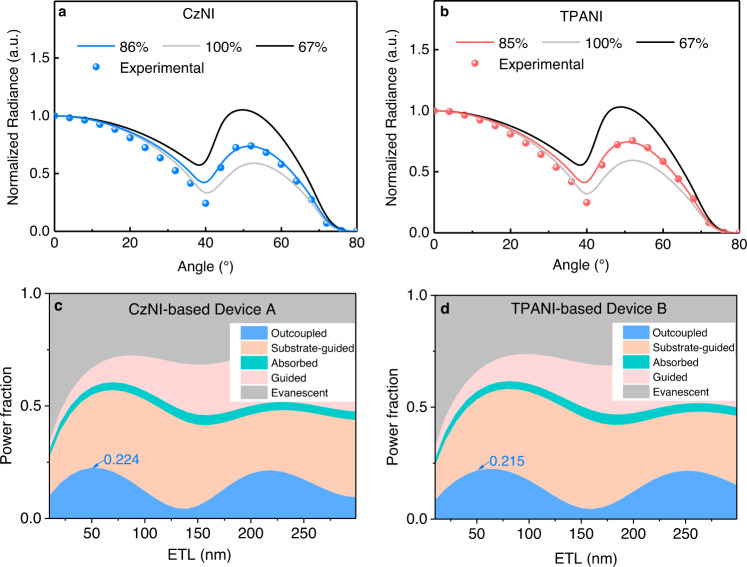
Horizontal dipole measurements together with optical simulation. *p*-Polarized angle-dependent PL radiance of neat film samples of **a** CzNI and **b** TPANI. Simulated photon distributions of all loss channels as a function of the electron transport layer (ETL) thickness for **c** CzNI-based device A and **d** TPANI-based device B. The loss channels include substrate-guided mode, absorbed mode, guided mode, and evanescent mode.

Since the EQE_max_ datum of an OLED correlates not only with the PLQY of the emitting layer and the electron-hole balance ratio (*γ*_e–h_), but also with the EUE and the light out-coupling efficiency (*η*_out_) (EQE = *γ*_e–h_ × *φ*_PL_ × EUE × *η*_out_)^14^, to gain insight into the reasons for the lower EQE_max_ of Device A than Device B, firstly, horizontal dipole measurements together with optical simulation were carried on CzNI and TPANI^34–36^. The results indicated that the neat film samples of CzNI and TPANI showed nearly identical horizontal dipole ratios (Θ_//_, 86% *vs*. 85%, vide Fig. 3), and hence the *η*_out_s of the CzNI-based Device A and the TPANI-based Device B are analogous (22.4% *vs*. 21.5%). Consequently, the probability that the efficiency difference between Devices A and B originates from the difference in their *η*_out_ can be ruled out.

**Fig. 4:**
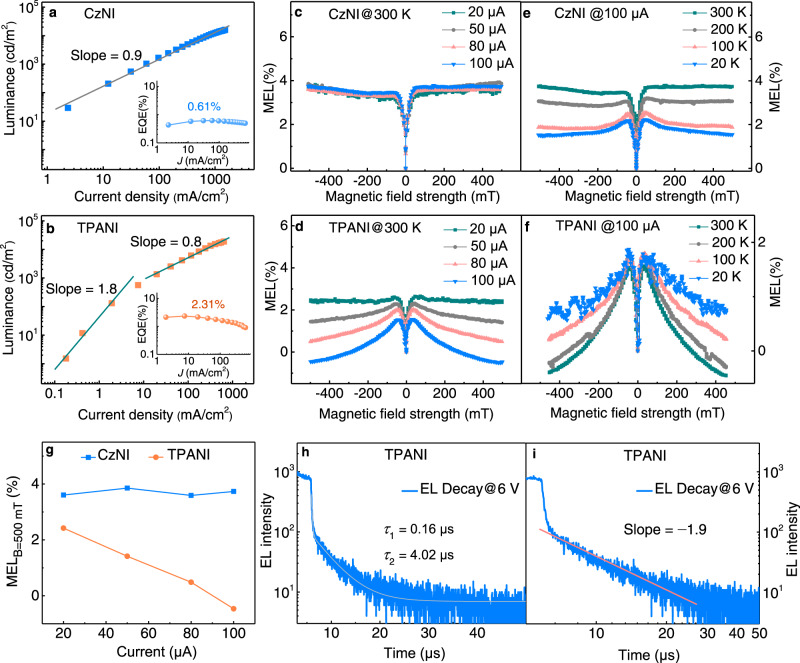
EL characteristics. **a**, **b** Luminance as a function of current density (insets: external quantum efficiency (EQE) as a function of current density (*J*) for devices). **c**, **d** Magneto-electroluminescence (MEL) response at different current density at room temperature. **e**, **f** MEL response at 100 μA at different temperatures. **g** The amplitudes of MEL response at 500 mT versus the applied current of the CzNI-based Device A and the TPANI-based Device B. **h** The fitting result of the single-logarithmic electroluminescence (EL) decay curve at 6 V, and **i** the fitting result of the double-logarithmic EL decay profile in a time range of 6–27 μs at 6 V, of the TPANI-based Device B (pulse width: 5 μs, *J* = 56.61 mA cm^−2^).

Based on the facts that the PLQY of CzNI is even higher than that of TPANI, the *η*_out_s of Devices A and B are quite similar, and the *γ*_e–h_s of Devices A and B should be analogous due to the very similar device structure, the higher EQE_max_ of the TPANI-based Device B than that of the CzNI-based Device A was tentatively ascribed to the worse triplet exciton utilization capability hence lower EUE of CzNI than TPANI. To validate this conjecture, magneto-electroluminescence (MEL) measurements were carried out on the two devices at room temperature (RT, 300 K) to probe the triplet harvesting mechanism of the two compounds^37–40^. As depicted in Fig. 4c, g, in a range of 20~100 μA, the CzNI-based Device A shows driving current-independent MEL profiles that increase sharply within the low-field regime (< 50 mT) then saturate in a higher *B*-field. For the TPANI-based Device B, however, the MEL profiles decrease gradually after reaching the maximum value, and this trend becomes more obvious under higher driving current (Fig. 4d, g). The quite different MEL profiles clearly demonstrate the quite different spin-dependent singlet–triplet transition processes in these two devices at RT, i.e., a TTA-*mediated* triplet utilization mechanism should exist in the TPANI-based device^37–40^, whereas the contribution from such mechanism is negligible in the CzNI-based device.

Given that TTA is essentially a bimolecular process, the luminance-current density (*L*-*J*) curve of Devices A and B were plotted in double-log form, and the slopes of the corresponding fitted lines were calculated. As shown in Fig. 4b, for the TPANI-based Device B, under relatively low excitation intensity conditions (*J* = 0.2–8.5 mA cm^−2^), the fitted line has a slope of ca. 1.8, indicative of the nearly quadratic dependence of luminance on current density in Device B; while at high excitation intensity (*J* = 20.2–686.7 mA cm^−2^), the slope turns to be ca. 0.8, which is almost linearly dependent. The observation of such two regimes in the *L*-*J* curve further confirmed that TTU contributes to the triplet exciton harvesting in this OLED^14,24,41–44^. On the contrary, in the entire current density region, the *L*-*J* curve of the CzNI-based Device A just showed a slope of ca. 0.9 (Fig. 4a), implying that the TTU mechanism should contribute insignificantly to the utilization of triplet excitons in this device^14^. Consistent with this deduction, long-lived delayed components were also discernable in the transient EL spectra of Device B^21,28,45^. As illustrated in Fig. 4h and Supplementary Fig. 19, after the Device B was pulsed off immediately, not only prompt components, but also delayed components with microsecond-scaled lifetime could be observed, signifying the contribution from TTU to EL^21,28,46,47^. Furthermore, as shown in Fig. 4i, the EL decay profile in double-log form can be linearly fitted with a slope of −1.9 at a time range of 6–27 μs, which fitted well with the TTA model^28^ as expressed in the Eq. (1) (where *I*_DF_ is the intensity of delayed EL induced by TTA-involved processes, *T*(*t*) is the T_1_ density at time of *t*, *γ*_TT_ is the rate constant of bimolecular TTA process of T_1_ excitons, see Supplementary Equations (1) ‒ (6) for details), indicating that it should be the TTA process that is responsible for the observed delayed EL emission in Device B (Fig. 4i)^14,21,22,28,48,49^. All these observations above verified that the TTU process contributes to the triplet exciton harvesting in the TPANI-based Device B.

1 $${I}_{{{{{{\rm{DF}}}}}}}\propto {[T(t)]}^{2}={\left(\gamma _{{{{{{\rm{TT}}}}}}}t+\frac{1}{[T(0)]}\right)}^{-2}$$

**Fig. 5:**
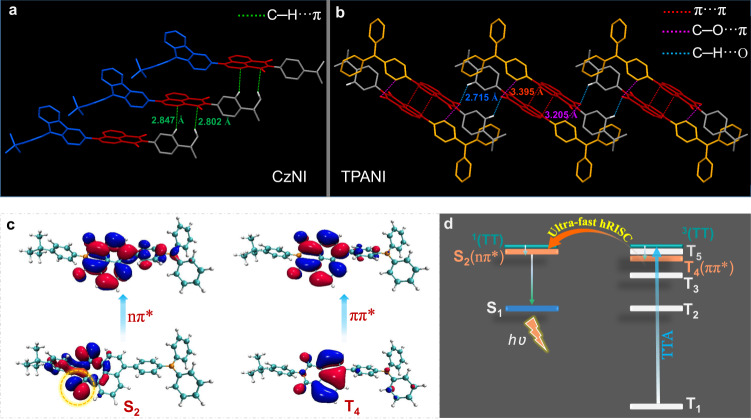
Molecular packing and theoretical calculation results. **a**, **b** Molecular packing pattern in single crystal samples of CzNI and TPANI. **c** NTO pairs of TPANI for the S_2_ and T_4_ states. **d** Proposed mechanism for the nπ*-involved TTA-*m*-hRISC in TPANI.

Since the T_1_ states of CzNI and TPANI both reside on their NI units (vide Supplementary Figs. 8 and 11), we speculated that the difference of TTU effect between the two compounds might originate from their differences in intermolecular stacking. Hence, the packing interactions and molecular alignment of CzNI and TPANI in their single crystal samples were analyzed. As shown in Fig. 5a, in CzNI crystals, the NI units were found to isolate from each other, and no regularly arranged dimers were discernable. In sharp contrast, TPANI displayed a well-defined parallel π-π stacking (Fig. 5b), which is probably attributable to the less steric bulk of TPA unit in comparison to Cz moiety in CzNI. As a result, there exists distinct dimeric face-to-face π-stacking between two planar NI segments (distance: 3.395 Å; π-overlap: ~50%), which may trigger rapid triplet diffusion of the NI-located T_1_ ‘cold’ excitons and hence strengthen the TTU process in TPANI^20,49^. Consequently, the more significant TTA effect of TPANI than CzNI may stem from the more effective intermolecular spatial proximity of its NI units^20,49^.

Since TTA response is sensitive to not only the applied current, but also the temperature, strong temperature-dependence should be observed in TTA-mediated MEL. Consistent with this deduction, with decreasing temperature from 300 K to 20 K, TTA-featured MEL profiles appear gradually in the CzNI-based Device A (Fig. 4e). Note that this low temperature-boosted TTA effect is commonly observed in OLEDs^37^, since the non-radiative decay of triplet excitons can be effectively suppressed at low temperature, leading to increased triplet exciton density that facilitates the occurrence of TTU. However, for the TPANI-based Device B, an abnormal low temperature-weakened TTA effect was discernable (Fig. 4f), implying that the triplet harvesting mechanism of TPANI should involve not only a TTA process, but also a thermal activation process. Inferred from the data that the experimentally determined $\Delta {E_{{{{\rm{S}}}}_{1}-{{{{\rm{T}}}}_{1}}}}$ of TPANI is 0.56 eV (Table 1), and the calculated $\Delta {E_{{{{\rm{T}}}}_{2}-{{{{\rm{S}}}}_{1}}}}$ of TPANI is ‒0.002 eV, the T_1_ → S_1_ RISC process should not be the dominant pathway to harvest triplet excitons, while the T_2_ → S_1_ *d*-hRISC process may not show a typical thermally activated character. Hence, we conjectured that additional endothermic hRISC processes may occur between higher-lying T_*n*_ (*n* > 2) and S_*m*_ (*m* > 1) states of TPANI, while such T_*n*_ excitons are formed through a triplet-channel TTA process using the ‘cold’ T_1_ excitons as the reactant.

**Fig. 6:**
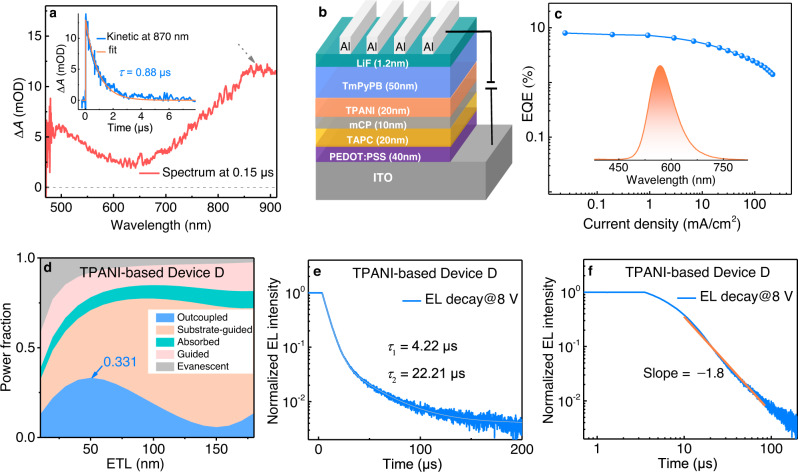
TA spectrum and EL characteristics. **a** ns-TA spectrum of TPANI in iodomethane under N_2_ atmosphere at RT (5 × 10^−4^ M, *λ*_ex_ = 355 nm, delayed time: 0.15 μs, OD: optical density). Inset: TA decay profile of TPANI at 870 nm. **b** Device structure and **c** external quantum efficiency (EQE) as a function of current density (*J*) for the TPANI-based optimal Device D. Inset: the EL spectrum of Device D (*J* = 220 mA cm^−2^). **d** Simulated photon distributions of all loss channels as a function of the electron transport layer (ETL) thickness for the TPANI-based Device D. **e** The fitting result of the single-logarithmic electroluminescence (EL) decay curve, and **f** the fitting result of the double-logarithmic EL decay profile in a time range of 10–90 μs of the TPANI-based Device D (pulse width: 500 μs, bias: 8 V).

To gain insight into the hRISC processes between higher-lying T_*n*_ (*n* > 2) and S_*m*_ (*m* > 1) states of TPANI, the electronic transition features of T_*n*_ (*n* > 2) and S_*m*_ (*m* > 1) states whose energies are lower than twice the T_1_ energy (~3.45 eV) together with the corresponding SOCMEs between T_*n*_ and S_*m*_ states were calculated for TPANI. The results indicated that for singlet excited states, only the S_2_-state energy meets the above requirement (vide Supplementary Fig. 14). Intriguingly, the S_2_ state of TPANI shows a ^1^nπ* transition nature that mainly located in its NI subunit (Fig. 5c), which, according to El-Sayed’s rule (Supplementary Fig. 13), can trigger quite effective SOC with the high-lying NI-involved ^3^ππ* excited states. In the case of triplet excited states, the calculated excitation energies of T_3_~T_5_ states of TPANI are all less than 3.45 eV (Supplementary Fig. 14 and Supplementary Table 10), yet only T_4_ and T_5_ states are calculated to be close to its S_2_ state ($\Delta {E_{{{{\rm{S}}}}_{2}-{{{{\rm{T}}}}_{4}}}}$: 0.10 eV; $\Delta {E_{{{{\rm{S}}}}_{2}-{{{{\rm{T}}}}_{5}}}}$: 0.03 eV). Excitingly, the T_4_ state is calculated to show a NI-predominant transition character (Fig. 5c), and hence a large SOCME can be boosted between the S_2_ and T_4_ states (calc. ${\mbox{SOCME}}_{({{{\rm{S}}}}_{2}-{{{\rm{T}}}}_{4})}$: 9.66 cm^−1^, vide Supplementary Table 11). Note that the presence of a NI-featured T_4_ state in TPANI is also corroborated by nanosecond transient absorption spectroscopy (ns-TA). As shown in Fig. 6a, there appears to be two broad absorption bands in the 700~910 nm region with absorption maxima of ca. 800 nm (1.55 eV) and 870 nm (1.42 eV), respectively. Taking into account that the two excited state absorption (ESA) bands are long-lived and highly sensitive to oxygen (Supplementary Fig. 15), both the two ESA bands can be attributed to the absorption of triplet states. Since according to Kasha’s rule, the T_*n*_ states (*n* > 1) often show a relatively short lifetime due to the fast IC processes, the TA signals of TPANI are assigned to T_1_ → T_*n*_ transitions. Based on the theoretical calculation results (vide Supplementary Table 10) that the T_4_ and T_5_ states of TPANI not only manifest a NI-dominated transition character which is similar to that of its T_1_ state, but also show a $\Delta {E_{{{{\rm{T}}}}_{1}{{{{\rm{T}}}}_{n}}}}$ of 1.62~1.69 eV that is close to the experimental findings (1.42~1.55 eV), we tentatively assigned the two absorption bands in 700-910 nm to T_1_ → T_5_ (~800 nm) and T_1_ → T_4_ (~870 nm) transitions, respectively. In addition, the computational result that T_4_ is lower-lying than S_2_ suggests that thermal activation is required to make the TTA-*m*-hRISC occur. By taking advantages of the concurrent small $\Delta {E_{{{{\rm{S}}}}_{2}-{{{{\rm{T}}}}_{4}}}}$ and very large ${\mbox{SOCME}}_{({{{\rm{T}}}}_{4}-{{{\rm{S}}}}_{2})}$, the T_4_ → S_2_ hRISC process in TPANI can be ultra-fast, and the calc. $k_{m{\mbox{-}}{{{\rm{hRISC}}}}({{{\rm{T}}}_{4}} \rightarrow {{{\rm{S}}}_{2}})}$ even exceeds 10^9 ^s^−1^ (Supplementary Table 11). Consequently, the triplet harvesting mechanism of TPANI should involve an ultra-fast TTA-*m*-hRISC process from its ^3^ππ*-featured T_4_ state to its ^1^nπ*-featured S_2_ state^50–52^.

Based on all these experimental and computational findings, a superior triplet harvesting mechanism model hidden in TPANI was unveiled. As depicted in Fig. 5d and Supplementary Fig. 14, directly injected ‘hot’ triplet excitons can be converted to singlet excitons via the fast T_2_ → S_1_ *d*-hRISC process; while the ‘cold’ T_1_ excitons, formed either through direct electro-injection or through IC process from T_*n*_ states, can also be effectively harnessed through triplet-channel TTA-*m*-hRISC with a cascade process of T_1_ + T_1_ → ^3^(TT) → T_4_ → S_2_ → S_1_. Here, the *m*-hRISC process is supposed to occur from the T_4_ state to the S_2_ state due to the experimentally observed endothermic features and the large calculated ${\mbox{SOCME}}_{({{{\rm{T}}}}_{4}-{{{\rm{S}}}}_{2})}$. Yet considering that the calculated T_5_ energy (3.42 eV) is also in the range of 3.40 − 3.45 eV (2 × T_1_), and the calculated $k_{{{{{\mathrm{hRISC}}}}}{({{{\rm{T}}}}_{5}-{{{\rm{S}}}}_{2})}}$ is as large as 1.6 × 10^8 ^s^−1^, the probability that a *m*-hRISC process occurs from the ^3^(TT) state to the S_2_ state could not be excluded^21^, leading to another possible cascade process of T_1_ + T_1_ → ^3^(TT) → S_2_ → S_1_. Nevertheless, regardless of whether the *m*-hRISC process occurs from the ^3^(TT) state or the T_*n*_ state, two ‘cold’ T_1_ excitons will be converted into one S_1_ exciton via the triplet-channel of TTU on the premise that the SOC between T_*n*_/^3^(TT) and S_m_ is relatively strong. Therefore, owing to the much more effective utilization of triplet excitons, the TPANI-based OLED shows a significantly higher EL performance than the CzNI-based reference device. In fact, upon further optimization on the device structure [ITO/PEDOT:PSS (40 nm)/TAPC (20 nm)/mCP (10 nm) /CzNI or TPANI (20 nm)/TmPyPB (50 nm)/LiF (1.2 nm)/Al (120 nm)], we fabricated a CzNI-based Device C and a TPANI-based Device D, respectively (vide Fig. 6b, c and Supplementary Fig. 20). Similarly, the EQE_max_ of the TPANI-based optimal Device D is much higher than that of the CzNI-based reference Device C (7.82% *vs*. 4.06%, vide Table 2). Additional optical simulation experiments revealed that the *η*_out_ of the Device C is 29.7% (Supplementary Fig. 22a), which is only slightly lower than that of the Device D (33.1%, Fig. 6d). Based on the PLQY datum of the neat film sample of CzNI (61.9%), the EUE_max_ of the CzNI-based Device C was calculated to be 22.1%, which is significantly lower than that of the TPANI-based Device D (46.7%).

**Table 2:** Summary of EL performance of the CzNI- and TPANI-based non-doped OLEDs

Device	Emitter	*V*_on_^a^ (V)	*L*_max_^b^ (cd/m^2^)	CIE_(x,y)_^c^	*λ*_EL,max_^d^ (nm)	EQE_max_^e^ (%)	CE_max_^f^ (cd/A)
A	CzNI	2.2	25430	0.22, 0.49	504	0.61	1.76
B	TPANI	3.2	18690	0.44, 0.52	560	2.31	7.44
C	CzNI	3.6	2905	0.27, 0.52	510	4.06	11.70
D	TPANI	4.0	9831	0.46, 0.52	566	7.82	22.09

^a^Turn-on voltage.

^b^Maximum luminance.

^c^Commission International de I’Eclairage 1931 coordinates.

^d^EL maximum.

^e^Maximum external quantum efficiency.

^f^Maximum current efficiency.

Since the much superior EL performance of the TPANI-based Device B than the CzNI-based Device A was demonstrated to stem from the better TTA-involved triplet exciton utilization capability of TPANI than CzNI, the much better EL performance of the Device D than the Device C may also originate from the better TTA-involved triplet harvesting in TPANI than in CzNI. This conjecture was verified through further transient EL measurements. As illustrated in Fig. 6e, Supplementary Figs 22 and 23, although both Devices C and D exhibited microsecond-scaled delayed components when the electric excitation was pulsed off, only the double-logarithmic EL decay profile of Device D could be linearly fitted with a slope of −1.8 in the 10–90 μs time region (Fig. 6f), verifying that triplet excitons can be harvested through TTA-involved processes in this device. In the case of Device C, however, only a slope of −1.0 could be observed in the similar time region (Supplementary Fig. 22d), excluding the significant TTA-involved triplet exciton utilization in this device. All these observations corroborated that the TTU-involved triplet exciton utilization is also more significant in the TPANI-based Device D than in the CzNI-based Device C.

### Triplet dynamics in TPANI-based device

Based on the experimental findings that the EUE_max_ of the TPANI-based Device D should be no less than 46.7%, which is higher than the spin statistical limit of TTA-OLED whose singlet and triplet TTA channels are both opened (40.0%, vide Fig. 1b), it can be deduced that additional TTA-*m*-hRISC process and/or direct hRISC (*d*-hRISC) process (from T_2_ to S_1_) should contribute to the triplet harvesting in this device. To decipher the triplet dynamics in Device D, the delayed component was demerged from the transient EL profile, and the relative composition (*I*_delay_/*I*_steady_) was calculated to be ca. 29% (Supplementary Fig. 24). Taking into consideration that generally, the rate constant of a hRISC process (*k*_hRISC_) is larger than that of a TTA process (*k*_TTA_)^7^, here the TTA step was assumed to be the rate determining step of the TTA-*m*-hRISC process. Hence in Device D, *d*-hRISC was supposed to contribute insignificantly to the microsecond-scaled delayed EL, and the delayed component is mainly resulted from TTA-involved processes, both singlet-channeled TTA and triplet-channeled TTA-*m*-hRISC. If there is no direct hRISC process contributing to EL in Device D, through the formula of *I*_delay_/*I*_steady_ = *η*_DF_/(*η*_S_ + *η*_DF_) [where *η*_S_ is the proportion of electrically generated singlet excitons (25.0% for small molecules), and *η*_DF_ is the proportion of singlet excitons generated via TTA-involved processes, i.e., *η*_TTA_ + *η*_TTA-*m*-hRISC_]^46,53^, the *η*_DF_ in Device D was calculated to be ca. 10.2%. Based on the fact that the sum of *η*_S_ and *η*_DF_ (35.2%) is much lower than the EUE_max_ (46.7%) of Device D, it can be deduced that the *d*-hRISC process should actually also contribute to the triplet utilization in this device. In this situation, according to the updated formula *I*_delay_/*I*_steady_ = *η*_DF_/(*η*_S_ + *η*_DF_ + *η*_*d*-hRISC_), the *η*_DF_ in Device D was re-calculated to be ca. 13.5% (29% × 46.7%), and thus the corresponding singlet exciton generation proportion (*η*_*d*-hRISC_) from *d*-hRISC process was calculated to be 8.2%.

**Fig. 7:**
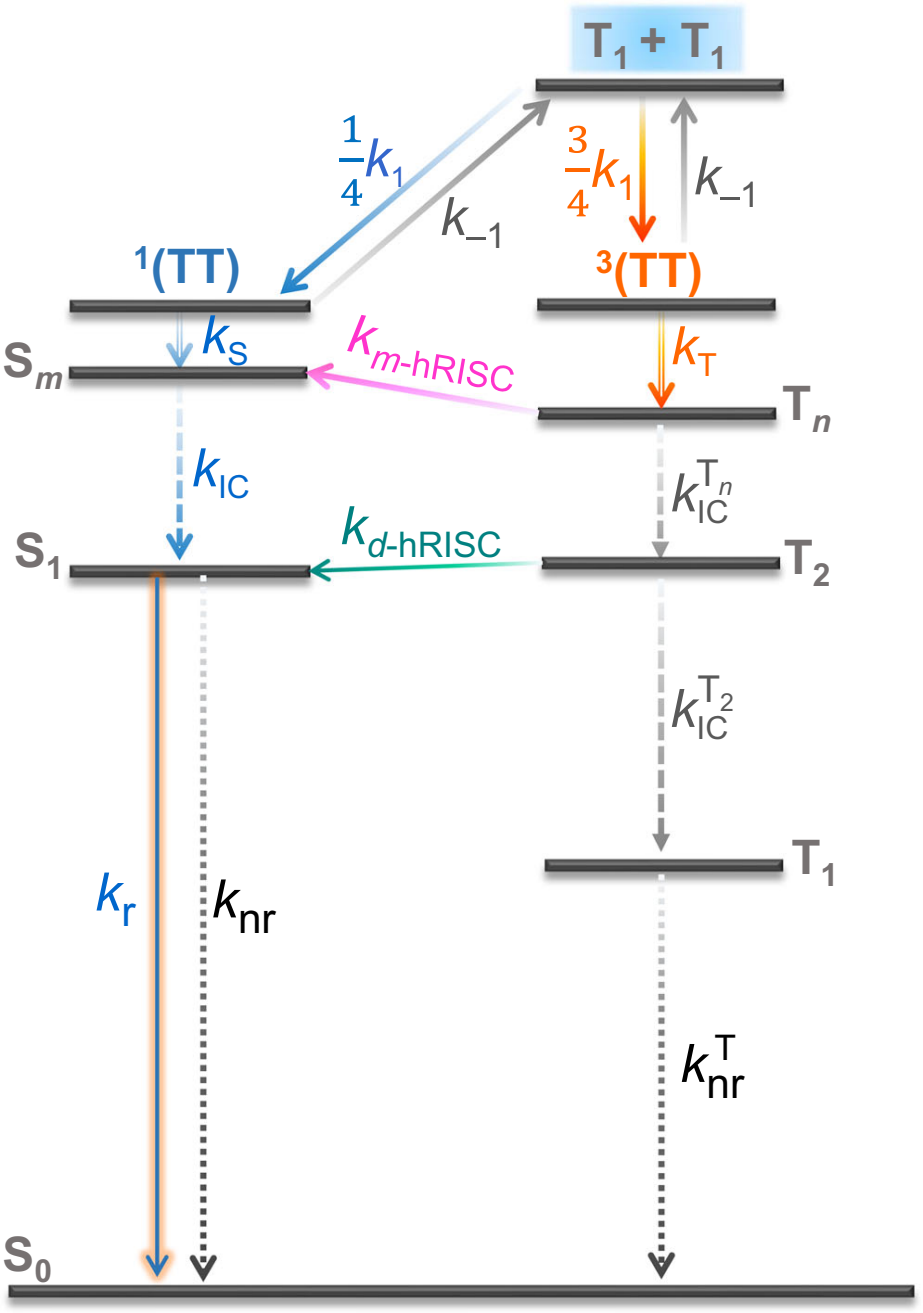
Schematic illustration of triplet dynamics. Detailed triplet exciton dynamics diagram in the TPANI-based Device D.

To gain insight into the contribution of TTA-*m*-hRISC process to the triplet utilization in Device D, the exciton generation/conversion processes in this device was schematically illustrated (vide Fig. 7), and the corresponding exciton dynamics can be expressed as follows:

2 $$\frac{{{{{{\rm{d}}}}}}[{{{{{{\rm{S}}}}}}}_{1}]}{{{{{{\rm{d}}}}}}{{t}}}=\frac{1}{4}G+0.082G-({k}_{{{{{{\rm{r}}}}}}}+{k}_{{{{{{\rm{nr}}}}}}})[{{{{{{\rm{S}}}}}}}_{1}]+{k}_{{{{{{\rm{IC}}}}}}}[{{{{{{\rm{S}}}}}}}_{{{{{m}}}}}]$$

3 $$\frac{{{{{{\rm{d}}}}}}[{{{{{\rm{T}}}}}}_{1}]}{{{{{{\rm{d}}}}}}{{t}}}=	\, \frac{3}{4}G-0.082G-2\frac{1}{4}{k}_{1}{[{{{{{{\rm{T}}}}}}}_{1}]}^{2}-2\frac{3}{4}{k}_{1}{[{{{{{{\rm{T}}}}}}}_{1}]}^{2}-{k}_{{{{{{\rm{nr}}}}}}}^{{{{{{\rm{T}}}}}}}[{{{{{{\rm{T}}}}}}}_{1}] \\ 	+{k}_{{{{{{\rm{IC}}}}}}}^{{{{{{{\rm{T}}}}}}}_{2}}[{{{{{{\rm{T}}}}}}}_{{{{{n}}}}}]+2{k}_{-1}[{\,\!}^{1}({{{{{\rm{TT}}}}}})]+2{k}_{-1}[{\,\!}^{3}({{{{{\rm{TT}}}}}})]$$

4 $$\frac{{{{{{\rm{d}}}}}}[{\!\,}^{1}({{{{{\rm{TT}}}}}})]}{{{{{{\rm{d}}}}}}{{t}}}=\frac{1}{4}{k}_{1}{[{{{{{{\rm{T}}}}}}}_{1}]}^{2}-{k}_{-1}[{\,\!}^{1}({{{{{\rm{TT}}}}}})]-{k}_{{{{{{\rm{S}}}}}}}[{\!\,}^{1}({{{{{\rm{TT}}}}}})]$$

5 $$\frac{{{{{{\rm{d}}}}}}[{\!\,}^{3}({{{{{\rm{TT}}}}}})]}{{{{{{\rm{d}}}}}}{{t}}}=\frac{3}{4}{k}_{1}{[{{\rm{T}}}_{1}]}^{2}-{k}_{-1}[{\!\,}^{3}({{{{{\rm{TT}}}}}})]-{k}_{{{{{{\rm{T}}}}}}}[{\!\,}^{3}({{{{{\rm{TT}}}}}})]$$

6 $$\frac{{{{{{\rm{d}}}}}}[{{{{{{\rm{T}}}}}}}_{{{{{n}}}}}]}{{{{{{\rm{d}}}}}}{{t}}}={k}_{{{{{{\rm{T}}}}}}}[{\!\,}^{3}({{{{{\rm{TT}}}}}})]-{k}_{m-{{{{{\rm{hRISC}}}}}}}[{{{{{{\rm{T}}}}}}}_{{{{{n}}}}}]-{k}_{{{{{{\rm{IC}}}}}}}^{{{{{{{\rm{T}}}}}}}_{{n}}}[{{{{{{\rm{T}}}}}}}_{{{{{n}}}}}]$$

7 $$\frac{{{{{{\rm{d}}}}}}[{{{{{{\rm{S}}}}}}}_{{{{{{{m}}}}}}}]}{{{{{{\rm{d}}}}}}{{t}}}={k}_{{{{{{\rm{S}}}}}}}[{\!\,}^{1}({{{{{\rm{TT}}}}}})]+{k}_{m-{{{{{\rm{hRISC}}}}}}}[{{{{{{\rm{T}}}}}}}_{{{{{n}}}}}]-{k}_{{{{{{\rm{IC}}}}}}}[{{{{{{\rm{S}}}}}}}_{{{{{m}}}}}]$$

where [S_1_], [T_1_], [T_*n*_], [S_*m*_], [^1^(TT)], [^3^(TT)] are the densities of the S_1_, T_1_, T_*n*_, S_*m*_ excitons, and the singlet-featured and triplet-featured intermediate states in sequence; *G* is the term for exciton generation; *k*_1_ and *k*_*−*1_ are the rate constants of the generation of TT pair via the collision of two T_1_ excitons and the dissociation of TT pair respectively; *k*_S_ and *k*_T_ are the rate constants of internal conversion (IC) process from ^1^(TT) and ^3^(TT) intermediate states to S_*m*_ and T_*n*_ respectively; *k*_*m*-hRISC_ is the rate constant of hRISC process from T_*n*_ to S_*m*_ in the TTA-*m*-hRISC process; *k*_IC_ and ${k}_{{{{{{\rm{IC}}}}}}}^{{{{{{{\rm{T}}}}}}}_{{n}}}$ are the rate constants of IC processes from S_*m*_ to S_1_ and from T_*n*_ to T_2_, respectively; *k*_nr_ and ${k}_{{{{{{\rm{nr}}}}}}}^{{{{{{\rm{T}}}}}}}$ are the rate constants of non-radiative processes from S_1_ to S_0_ and from T_1_ to S_0_, respectively. Through the solution of Eq. (2–7) (vide Supplementary Equations (7–19) for details), the ratio of *η*_TTA-*m*-hRISC_ to *η*_TTA_ can be expressed as follows:

8 $$\frac{{\eta }_{{{{{{\rm{TTA}}}}}}-m-{{{{{\rm{hRISC}}}}}}}}{{\eta }_{{{{{{\rm{TTA}}}}}}}}=\frac{3\,{k}_{m-{{{{{\rm{hRISC}}}}}}}}{{k}_{{{{{{\rm{IC}}}}}}}^{{{{{{{\rm{T}}}}}}}_{{n}}}+{k}_{m-{{{{{\rm{hRISC}}}}}}}}$$

Considering that the *k*_*m*-hRISC_ of TPANI is calculated to be as large as 2.1 × 10^9^ s^−1^, and meanwhile the relatively large calculated $\Delta {E_{({{{\rm{T}}}}_{2}{{{\rm{T}}}}_{4})}}$ (~0.6 eV) may lead to a relatively slow T_4_ → T_2_ IC process, it is assumed that *k*_*m*-hRISC_ » ${k}_{{{{{{\rm{IC}}}}}}}^{{{{{{{\rm{T}}}}}}}_{{n}}}$, and thus the *η*_TTA-*m*-hRISC_/*η*_TTA_ is 3. Consequently, the total *η*_DF_ of 13.5% in Device D can be divided into two parts: *η*_TTA_ of ca. 3.4% and *η*_TTA-*m*-hRISC_ of ca. 10.1%. All these results manifested that even if ‘cold’ T_1_ excitons can be generated in hRISC-OLEDs, through either direct electrical injection or IC process from the higher-lying T_*n*_ states, they can be effectively harvested via the TTA-*m*-hRISC process.

## Discussion

In summary, through comparative studies on two naphthalimide-based compounds CzNI and TPANI, we unveiled that the ‘cold’ T_1_ excitons in hRISC materials can be utilized effectively through the TTA-*m*-hRISC mechanism (T_1_ + T_1_ → ^3^(TT) → T_*n*_ → S_*m*_ → S_1_) if they are endowed with triplet-triplet upconversion capability. It is noteworthy that the T_*n*_ → S_*m*_ *m*-hRISC process in TPANI shows a ^3^ππ*→^1^nπ* instead of a ^3^ππ*→^1^ππ* transition character. By taking advantage of the permanent orthogonal orbital transition effect of ^3^ππ*→^1^nπ*, a SOCME of near 10 cm^−1^ can be acquired, and hence an ultra-fast *m*-hRISC process with a *k*_*m*-hRISC_ exceeding 10^9 ^s^−1^ can be realized in TPANI, without the need of constructing orthogonal D-A diads bearing anthracene derivative subunits. Moreover, as the S_1_ state of TPANI shows HLCT characters, a relatively large *k*_f_ of 10^8 ^s^−1^ is readily acquired. Benefiting from this mechanism, despite of the relatively low PLQY of TPANI (50.6%), a decent EQE_max_ of 7.8% and EUE_max_ of at least 46.7% are acquired in a TPANI-based OLED. These results present a method to facilely access hRISC materials, and can greatly extend the design rationales for high-performance OLED materials.

## Methods

### Materials

Unless otherwise described, all reagents and anhydrous solvents were purchased from commercial sources and used as received. All the solvents used in photophysical measurements were of analytical grades and freshly distilled prior to use. CzNI and TPANI were purified through three times recrystallization followed by vacuum sublimation.

### General measurements

^1^H NMR and ^13^C NMR spectra were recorded on a Bruker AVANCE II-400 MHz spectrometer at 400 and 100 MHz in CDCl_3_, respectively. Tetramethylsilane (TMS) was used as an internal standard. All chemical shift data were reported in the standard *δ* notation of parts per million (ppm). Splitting patterns were designed as follows: s (singlet), d (doublet), t (triplet), and m (multiplet). Cyclic voltammetry (CV) characterization was performed on a LK2010 electrochemical workstation, and this measurement was calibrated with an internal standard ferrocene/ferrocenium (Fc/Fc^+^) redox system. High-resolution mass spectra were measured on a Q-TOF Premier ESI mass spectrometer (MS, Micromass, Manchester, UK). UV-visible spectra were measured on a Shimadzu UV-3600 spectrophotometer. Steady-state photoluminescence (PL) spectra at room temperature (RT) and PL quantum efficiency data were measured on Horiba Jobin Yvon Fluorolog-3 fluorescence spectrophotometer. Delayed emission spectra with different delay times at 77 K were measured on a Horiba Jobin Yvon Fluoromax fluorescence spectrophotometer. Transient PL decay profiles at RT under N_2_ atmosphere were recorded on a Horiba Jobin Yvon FluoroHub-B equipped with a single photon counting controller. Single-crystal X-ray diffraction data were obtained on a Bruker D8 Venture X-ray single-crystal diffractometer. Single crystal samples of CzNI and TPANI were obtained by slow evaporation of saturated solution from mixed solvents (dichloromethane and ethanol) under room temperature. The crystallographic data for CzNI and TPANI reported here have been deposited in the Cambridge Structural Database with CCDC numbers 2152120 and 2152121, respectively.

### Sub-nanosecond transient absorption spectroscopy measurements

The absorption and lifetime of the emissive and non-emissive transient species can be fully characterized by using this transient absorption spectroscopy technique. These measurements of compound TPANI were done in dilute iodomethane solutions (5 × 10^−4 ^M) on the EOS (Ultrafast Systems, USA) system. The instrument response function (IRF) of this setup is determined to be ~100 ps. For this investigation, a 355 nm excitation (pulse duration 1 ns, pulse energy 7 μJ, repetition rate 1 kHz) was used to pump the molecules to the excited state and the white-light continuum (WLC) probe pulses (350-910 nm) was used for probing the excited state. The temporal and spectral profiles of the pump-induced differential absorbance of the probe light (Δ*A*) are visualized by an optical fiber-coupled multichannel spectrometer (with a CMOS sensor) and further analyzed by the Surface Xplorer (SX) software.

### Computational method

The initial geometry of CzNI or TPANI was extracted from their single crystal structures and then further optimized. The geometry of the ground state (S_0_) was optimized at density functional theory (DFT) level using B3LYP hybrid functional and 6-31 G(d) basis. The geometry of the lowest triplet state (T_1_) was optimized using spin-unrestricted CAM-B3LYP hybrid functional and 6-31 G(d) basis. For other singlet and triplet excited states, the geometries were optimized using time-dependent DFT (TD-DFT) method with CAM-B3LYP density functional. Furthermore, the energy of individual excited state was obtained using LC-*ω*PBE hybrid functional with basis of 6-31+G(d) based on their respective optimized molecular geometries accordingly^54^, where *ω* parameter for long-range correction was optimized and determined as 0.1805 and 0.1849 for CzNI and TPANI, respectively by taking advantage of optDFTw program^55–57^. The calculations described above were performed using Gaussian 09 software package^58^. In order to obtain the SOCME values for evaluating the RISC rate constants, PySOC^59–61^, a procedure combined with Gaussian 09, was subsequently used to calculate SOCME between triplet excited states and singlet excited state of TPANI. The solvent effect in all the calculations was conducted using the polarizable continuum model (toluene).

### Device fabrication, characterization, and optical simulation

PEDOT:PSS films (for Devices C and D) were spin-coated on pre-cleaned ITO glass substrates and annealed at 120 °C for 20 min, then the hole transport layers (HTLs) and the light-emitting layers (EMLs) materials were evaporated onto the PEDOT:PSS substrate in sequence. After that, electron transport layer (ETL) was evaporated onto the active layer. Finally, the LiF was deposited to act as electron injection layer, and Al was deposited to serve as metal cathode. In order to prevent degradation and emission quenching caused by oxygen and water, all the above operations are performed in a nitrogen atmosphere or a vacuum state (1 × 10^−4 ^Pa), and the OLED is encapsulated before characterization. The current–voltage–luminance ( *J*–*V*–*L*) characteristics were measured with a Keithley 2400 source measurement and a Lambertian distribution. The EL spectra and CIE coordinates were obtained by Photo Research PR735 optical analyzer. The EQEs were calculated on the premise of a Lambertian distribution by using the EL spectra, luminance, and current densities. All the measurements were carried out at room temperature under ambient conditions.

Angle-dependent photoluminescence was measured to obtain the horizontal dipole ratio (HDR, Θ//) of the light emission molecules in neat film state. *p*-polarized angle-dependent light emissions of films were measured by Fluxim. The HDR of thin films and light out-coupling efficiency of devices were then simulated by Setfos 5.1.

## Supplementary information

Supplementary Information

## Data Availability

The data supporting the findings of this study are available within the paper and the Supplementary Information. The X-ray crystallographic coordinates for structures reported in this study have been deposited at the Cambridge Crystallographic Data Centre (CCDC), under deposition numbers 2152120-2152121. These data can be obtained free of charge from The Cambridge Crystallographic Data Centre via www.ccdc.cam.ac.uk/data_request/cif. Source data are provided with this paper.

## Source data

Source Data
