# Supplementary material for: Ultra-fast triplet-triplet-annihilation-mediated high-lying reverse intersystem crossing triggered by participation of nπ*-featured excited states
Source: Nat Commun. 2022 Nov 12;13:6892. doi: 10.1038/s41467-022-34573-2 (PMC9653383; doi:10.1038/s41467-022-34573-2)
Supplement: Supplementary file 1 — Supplementary Information [file 41467_2022_34573_MOESM1_ESM.pdf]

Supplementary Information

Ultra-fast Triplet-triplet-annihilation-mediated High-lying Reverse Intersystem Crossing Triggered by Participation of nπ*-featured Excited States

Yanju Luo,[1,2] Kai Zhang,[3] Zhenming Ding,[3] Ping Chen,[4]* Xiaomei Peng,[5] Yihuan Zhao,[1] Kuan Chen,[1] Chuan Li,[1] Xujun Zheng,[1] Yan Huang,[1] Xuemei Pu,[1] Yu Liu,[3]* Shi-Jian Su,[5]* Xiandeng Hou,[1,2] and Zhiyun Lu[1]*

Supplementary Note 1. Synthetic procedures and characterization data

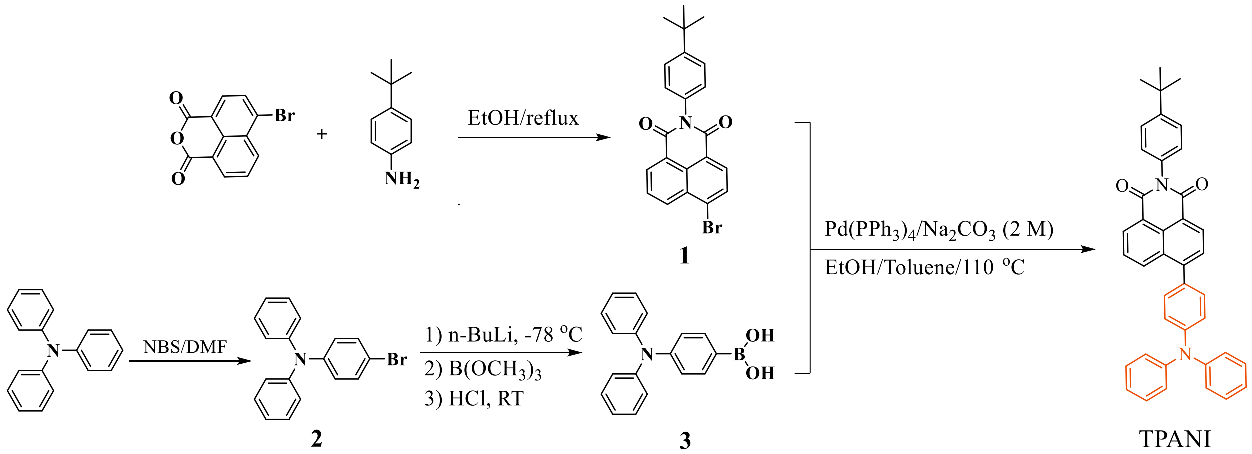

Supplementary Figure 1. **Synthetic route**. The detailed synthetic route to intermediates **1**, **2** and **3** as well as target compound TPANI.

The intermediates **1** and **2** as well as reference compound CzNI were synthesized according to reported procedure. [1, 2]

The general synthetic procedures of **3** and TPANI were as follows:

Synthesis of (4-(diphenylamino)phenyl)boronic acid (3)

A solution of *n*-C_4H_9Li [in hexane, 15 mL, 41.6 mmol] was added dropwise to a solution of **3** (6.0 g, 20.8 mmol) in dry THF (130 mL) at −78 ℃. After the addition was completed, the reaction mixture was stirred 1h at −78 ℃ and then allowed to warm to room temperature. The mixture was stirred for 24 h, and then quenched by aqueous HCl solution (27 ml, 2mol/L). The organic layer was separated, and the aqueous layer was extracted with EA (3 × 50 mL). The combined organic layers were washed with brine (3 ×40 mL) and dried over anhydrous $MgSO_4$, then filtered and concentrated in vacuum. Then recrystallization with n-hexane/CH_2Cl_2 to afford the product. Yield: 67%. Because of the instability of **3**, the crude product was not further purified.

Synthesis of 2-(4-(*tert*-butyl)phenyl)-6-(4-(diphenylamino)phenyl)-1*H*-benzo[*de*]isoquinoline-1,3(2*H*)-dione (TPANI)

A flask was charged with 1 (1.5 g, 3.67 mmol), 3 (1.1 g, 0.52 mmol), $Pd(PPh_3)_4$ (0.13 g, 0.016 mmol), aqueous $NaCO_3$ (100 mL, 2 mol L^{-1}) solution, ethanol (40 mL) and toluene (60 mL), and the reaction mixture was stirred at 110 °C for 7 h under argon. After the completion

of the reaction, the reaction mixture was cooled down, then poured into 60 mL water and extracted with CH_2Cl_2 (30 mL × 3). The resultant organic phase was washed with brine, and dried over anhydrous Na_2SO_4. After the solvent was removed, the residue was purified using column chromatography on silica gel employing petroleum ether/CH_2Cl_2 (1/4). Then recrystallization with n-hexane CH_2Cl_2/methanol to afford the greenish-yellow solid product. Yield: 57%. ^{1}H NMR (400 MHz, $CDCl_3$) δ (ppm): 8.72-8.63 (m, 2H), 8.47 (dd, J_1 = 8.4 Hz, J_2 = 0.8 Hz, 1H), 7.80-7.71 (m, 2H), 7.57 (d, J = 8.8 Hz, 2H), 7.40 (d, J = 8.4 Hz, 2H), 7.33 (t, J = 8.0 Hz, 4H), 7.27-7.21 (m, 8H), 7.11 (t, J = 7.2 Hz, 2H), 1.39 (s, 9H). ^{13}C NMR (100 MHz, $CDCl_3$) δ (ppm): 164.5, 164.7, 160.1, 151.4, 148.4, 147.4, 147.1, 133.1, 132.6, 131.8, 131.6, 131.3, 130.8, 130.1, 129.5, 129.2, 128.0, 128.0, 127.0, 126.5, 125.1, 123.7, 123.0, 122.5, 121.5, 34.7, 31.3. HRMS (ESI) m/z for $C_{40}H_{32}N_2O_2H^+$ [M + H]$^+$ calcd.: 573.2542, found: 573.2538; $C_{40}H_{32}N_2O_2Na^+$ [M + Na]$^+$ calcd.: 595.2361, found: 595.2356.

Supplementary Note 2. Photophysical characterization and theoretical calculation results

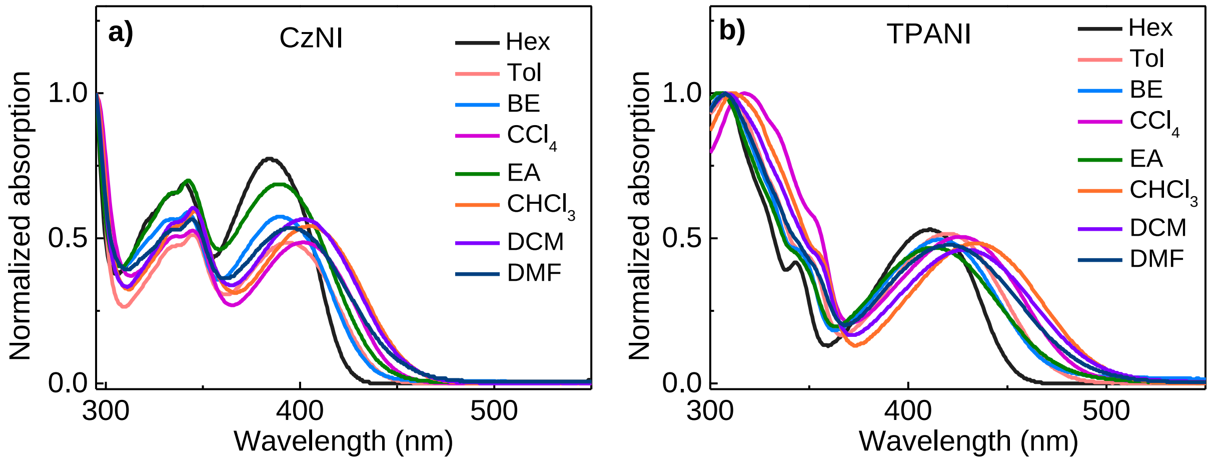

Supplementary Figure 2. Absorption spectra. Normalized absorption spectra (10^{-5} M) of a) CzNI and b) TPANI in solvents with different polarity. Hex: hexane; Tol: toluene; BE: butyl ether; EA: ethyl acetate; DCM: dichloromethane; DMF: *N*,*N*-Dimethylformamide.

υ_a-υ_f is the Stokes shift, $f(\varepsilon, n)$ is the orientational polarizability of solvents[3] given by

$$f(\varepsilon, n) = [\frac{\varepsilon - 1}{2\varepsilon + 1} - \frac{n^2 - 1}{2n^2 + 1}]$$

Where ε is the solvent dielectric constant and n is the solvent refractive index.

Supplementary Table 1. Photophysical data of CzNI and TPANI in dilute solution at room temperature.

				CzNI			TPANI		
Solvent	ε	n	$f(\varepsilon, n)$	λ_{abs} [a)] (nm)	λ_{em} [b)] (nm)	υ_a-υ_f (cm^{-1})	λ_{abs} [a)] (nm)	λ_{em} [b)] (nm)	υ_a-υ_f (cm^{-1})
Hex	1.9	1.375	0.0012	383	437	3226	410	470	3114
BE	3.08	1.399	0.096	391	481	4785	417	534	5254
EA	6.02	1.372	0.1998	389	524	6623	415	602	7485
$CHCl_3$	4.81	1.446	0.1482	405	534	5965	435	602	6377
DCM	8.93	1.424	0.2172	404	558	6831	430	645	7752
DMF	37	1.427	0.2757	397	602	8578	424	706	9421

[a)] λ_{abs} is the absorption peak at the long-wavelength side; [b)] λ_{em} is the emission peak.

Supplementary Table 2. Photophysical data of CzNI and TPANI in dilute solution at room temperature.

	CzNI		TPANI	
Solvent	λ_{em} (nm)	PLQY[a)] (%)	λ_{em} (nm)	PLQY[a)] (%)
Hex	437	83.6	470	84.1
BE	481	60.5	534	61.9
$CHCl_3$	534	58.9	602	59.7
EA	524	66.3	602	44.7
THF	521	65.8	607	40.9
DCM	558	62.8	645	32.4
DMF	602	0.31	706	<0.1

[a)] PLQY: relative PL quantum yield, determined using quinoline sulfate as the reference (PLQY: 44%).

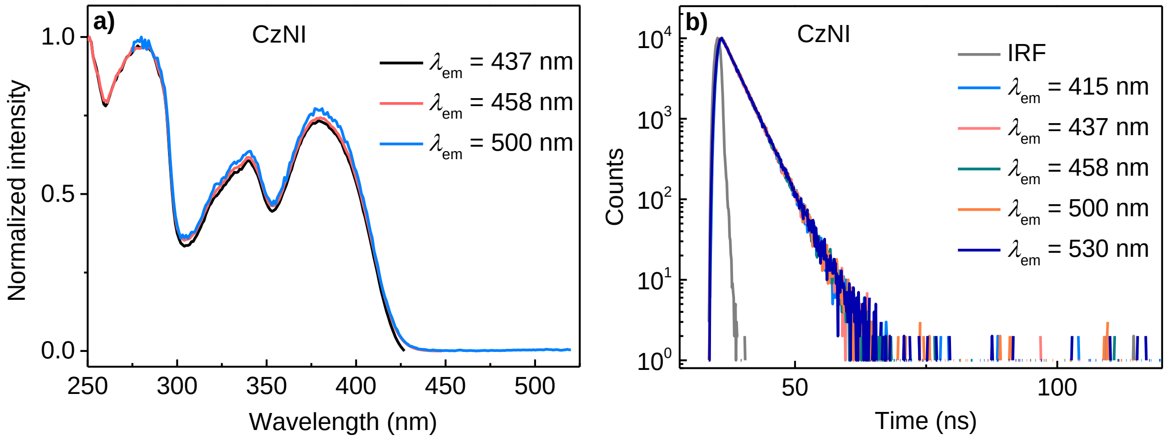

Supplementary Figure 3. Excitation spectra and transient PL profiles. a) Excitation spectra and b) transient PL curves of CzNI in the *n*-hexane solutions.

In nonpolar *n*-hexane, the double emission bands of CzNI (λ_{em} = 437 nm and 458 nm) are identified to be vibrational structures of its local singlet excited state (^{1}LE) rather than two different excited state species. This deduction has been verified by the excitation spectra and transient PL measurements. As shown in Supplementary Figure 3a, the two emission bands of the *n*-hexane solution of CzNI show identical excitation spectra, indicating that the two emission bands originate from the same excited state species rather than impurities. Furthermore, both the two emission bands show similar transient PL decay curves (Supplementary Figure 3b), indicating that the two bands originate from similar excited state species.

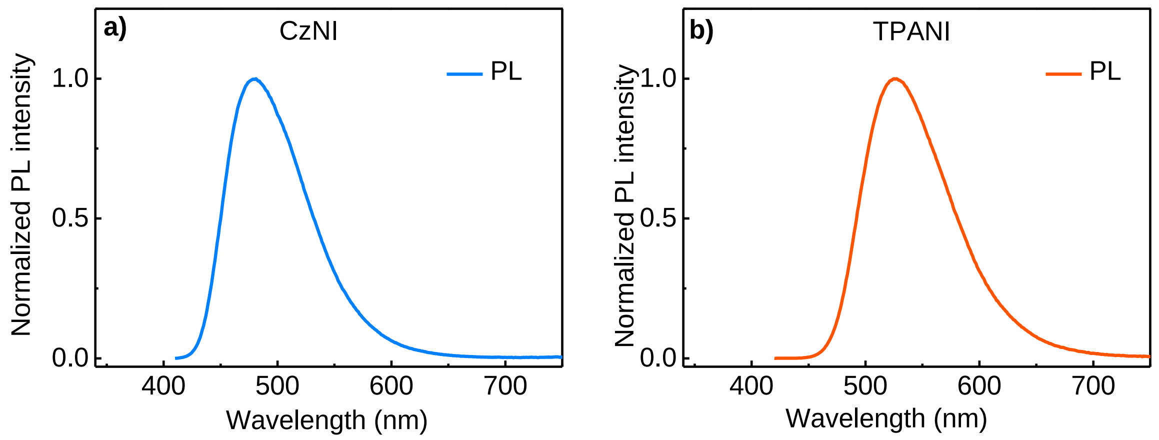

Supplementary Figure 4. PL spectra. Normalized Photoluminescence spectra of a) CzNI and b) TPANI in toluene (10^{-5} M).

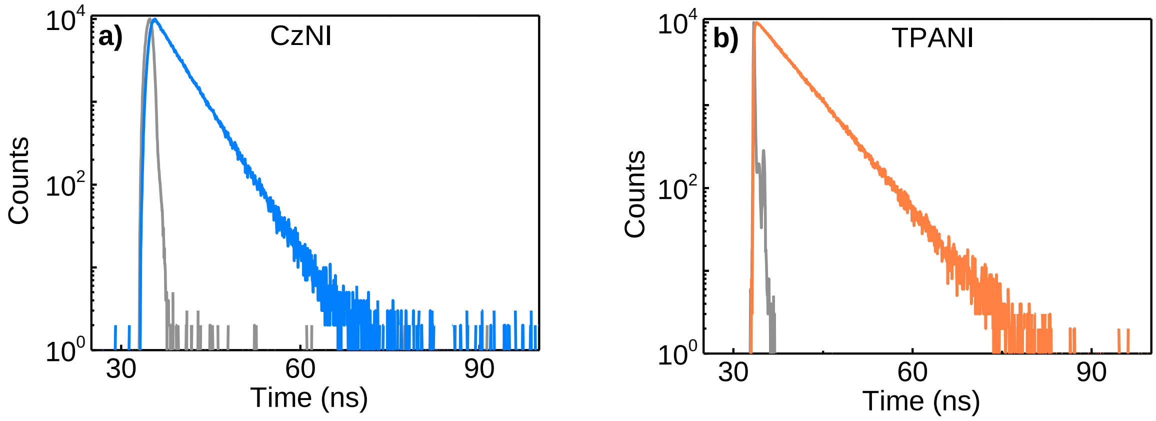

Supplementary Figure 5. Transient PL decay profiles. Transient PL decay curves of a) CzNI and b) TPANI in toluene solution under a N_2 atmosphere at RT (10^{-5} M, λ_{ex} = 370 nm).

Supplementary Table 3. Lifetime data of CzNI and TPANI in toluene solution under a N_2 atmosphere at RT (λ_{ex} = 370 nm).

Compound	λ_{em}	Lifetime	Weight	χ^2	PLQY	k_f
CzNI	473 nm	3.69 ns	100%	1.08	43%	1.1×10^8 s^{-1}
TPANI	520 nm	4.89 ns	100%	1.18	50%	1.0×10^8 s^{-1}

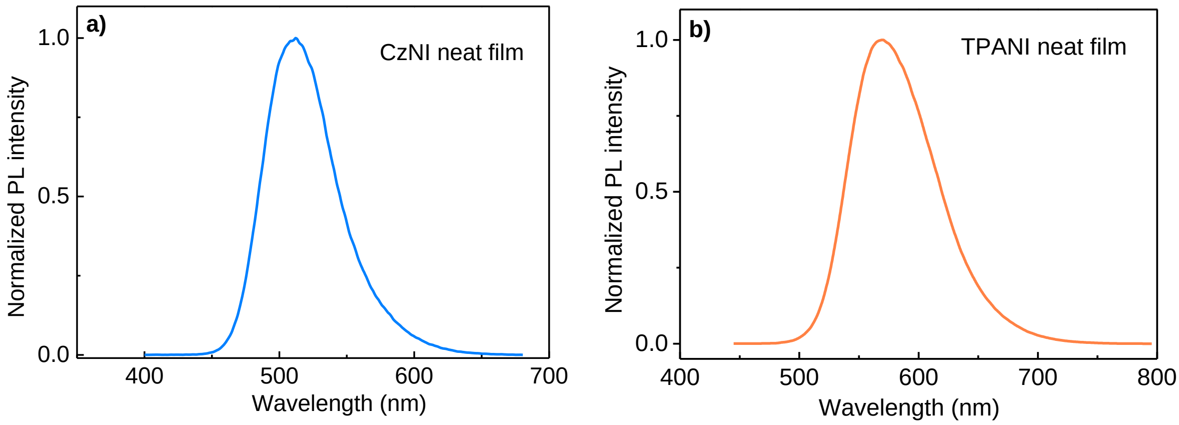

Supplementary Figure 6. PL spectra. Normalized Photoluminescence spectra of a) CzNI neat film (λ_{ex} = 390 nm) and b) TPANI neat film (λ_{ex} = 420 nm).

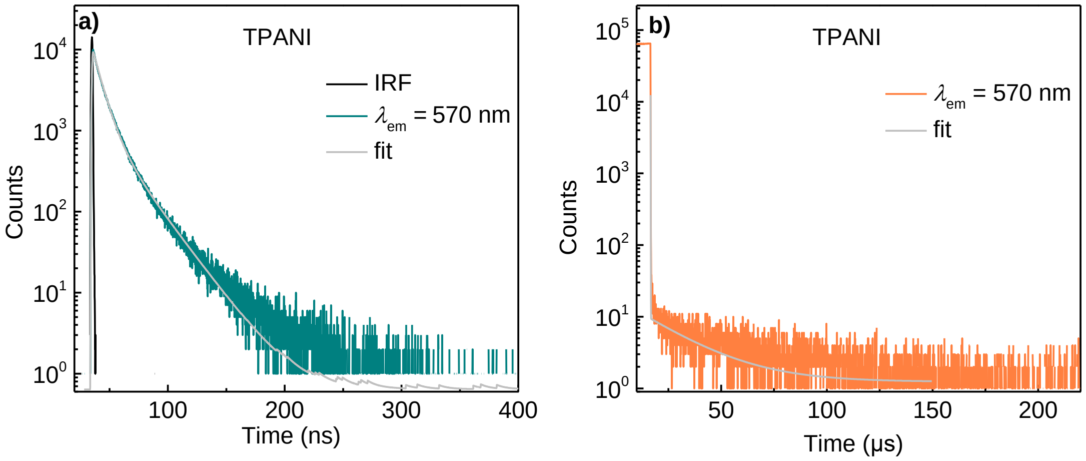

Supplementary Figure 7. Transient PL decay profiles. Transient PL decay curves of TPANI neat film at time-range windows of a) 400 ns and b) 340 μs respectively under a N_2 atmosphere at RT (λ_{ex} = 370 nm).

Supplementary Table 4. Lifetime data of TPANI neat film under a N_2 atmosphere at RT.

Measurement window	Lifetime	Weight	χ^2
400 ns	τ_1 = 7.26 ns<br>τ_2 = 22.38 ns	87%<br>13%	1.28
340 μs	τ_1 = 22.52 ns<br>τ_2 = 22.43 μs	99.9%<br>0.1%	1.04

As illustrated in Supplementary Figure 7 and Supplementary Table 4, not only prompt fluorescence (PF), but also delayed fluorescence (DF) component (lifetime: ~μs) can be clearly identified. Nevertheless, the relative weight of the DF component is very low, which may arise from the rather low triplet density under photo-excitation at room temperature.

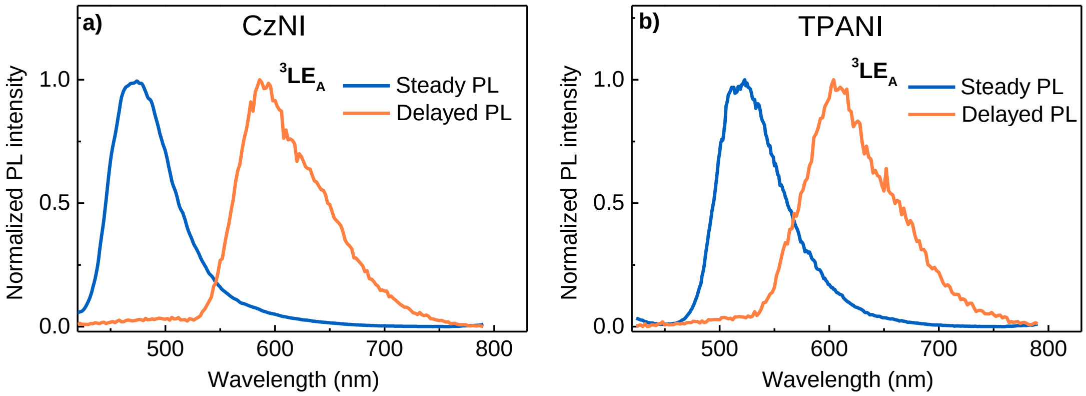

Supplementary Figure 8. Delayed emission spectra of solution samples. Delayed emission spectra with a delay time of 30 ms for a) CzNI and b) TPANI in iodoethane solutions (5×10^{-5} M, gate time: 41 ms) at 77 K.

After a relatively long delay time of 30 ms, the two emission bands (λ_{em} ~590 nm for CzNI, λ_{em} ~600 nm for TPANI) are still discernable, which differ from the corresponding steady PL in both shape and position, confirming their phosphorescence character.

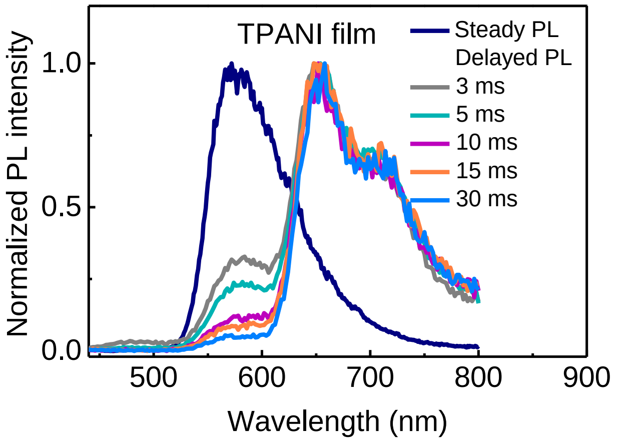

Supplementary Figure 9. Delayed emission spectra of film sample. Delayed emission spectra with different delay time (gate time: 41 ms) of TPANI neat film at 77 K (λ_{ex} = 420 nm).

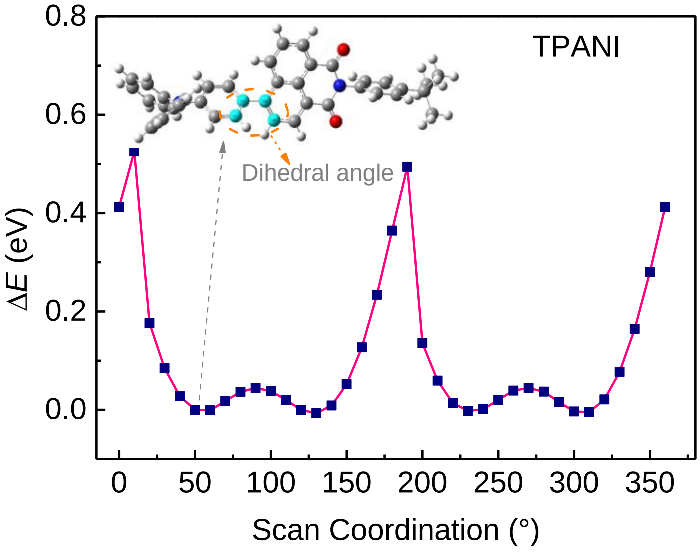

Supplementary Figure 10. Potential energy scan calculation of TPANI. Potential energy scan of TPANI conformations in the ground state calculated in the toluene solution at the CAM-B3LYP/6-31g(d) level. Scanned dihedral angle highlighted in orange.

A relaxed potential energy surface scan modelling for TPANI conformations in the ground state was conducted by progressively modulating the dihedral angle between the D and A units using toluene as the solvent. As shown in Supplementary Figure 10, for the lowest-energy conformation of TPANI, its dihedral angle is calculated to be ca. 50°, which is quite close to that observed in the single crystal sample (45°). Hence, the D-A dihedral angles for TPANI in toluene may be analogous to that in crystal.

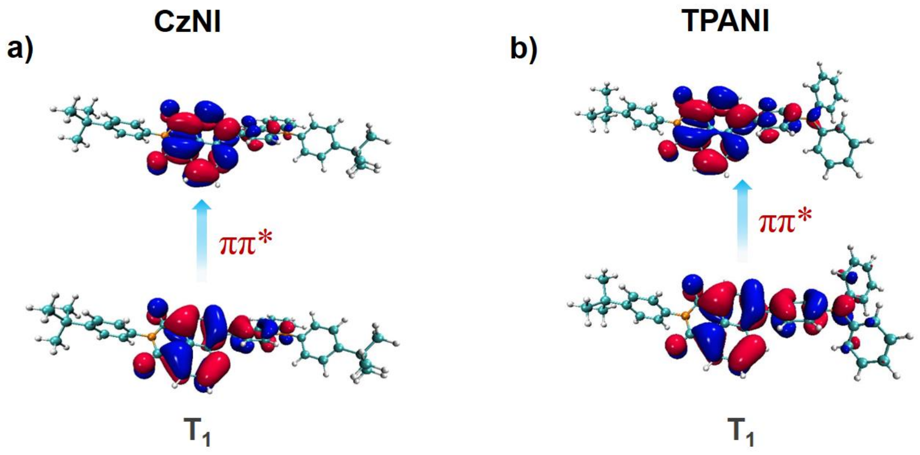

Supplementary Figure 11. Transition nature of CzNI and TPANI in T_1 states. The natural transition orbitals (NTO) of a) CzNI and b) TPANI in T_1 excited states revealed by TDDFT calculations by employing the LC-ωPBE functional with the 6-31+G(d) basis set.

Supplementary Table 5. Structure data of CzNI and TPANI single crystal.

	CzNI	TPANI
Empirical formula	$C_{44}H_{38}N_2O_2$	$C_{40}H_{32}N_2O_2$
Formula weight	626.76	572.67
Temperature/K	302.0	103.0
Crystal system	monoclinic	monoclinic
Space group	$P2_1/n$	C2/c
a/Å	15.1168(5)	30.275(2)
b/Å	8.4894(2)	15.7440(11)
c/Å	28.0109(10)	13.6204(8)
α/°	90	90
β/°	98.6780(10)	104.046(2)
γ/°	90	90
Volume/$Å^3$	3553.56(19)	6298.0(7)
Z	4	8
ρ_{calc}g/cm^3	1.172	1.208
μ/mm^{-1}	0.071	0.074
F(000)	1328.0	2416.0
Crystal size/mm^3	0.43 × 0.32 × 0.09	0.25 × 0.05 × 0.05
Radiation	MoKα (λ = 0.71073)	MoKα (λ = 0.71073)
2Θ range for data collection/°	4.824 to 55.04	4.9 to 55.186
Index ranges	$-19 \le h \le 19$, $-11 \le k \le 10$, $-36 \le l \le 36$	$-39 \le h \le 39$, $-19 \le k \le 20$, $-17 \le l \le 17$
Reflections collected	48695	55392
Independent reflections	8165 [R_{int} = 0.0980, R_{sigma} = 0.0727]	7215 [R_{int} = 0.1639, R_{sigma} = 0.0837]
Data/restraints/parameters	8165/21/439	7215/0/400
Goodness-of-fit on F^2	1.025	0.988
Final R indexes [I>=2σ (I)]	R_1 = 0.0709, wR_2 = 0.1914	R_1 = 0.0494, wR_2 = 0.0977
Final R indexes [all data]	R_1 = 0.1154, wR_2 = 0.2235	R_1 = 0.1126, wR_2 = 0.1175
Largest diff. peak/hole /e $Å^{-3}$	0.56/-0.39	0.15/-0.20

Supplementary Table 6. Fractional atomic coordinates ($\times10^4$) and equivalent isotropic displacement parameters ($Å^2\times10^3$) for CzNI. U_{eq} is defined as 1/3 of the trace of the orthogonalised U_{IJ} tensor.

**Atom**	***x***	***y***	***z***	**U(eq)**
O1	6671.5(17)	10919(3)	5595.2(8)	85.5(7)
O2	7501.5(15)	9516(2)	7158.3(7)	74.9(6)
N1	7108.6(13)	10192(2)	6374.5(7)	49.6(5)
N2	2939.7(14)	38(2)	6362.6(8)	53.8(5)
C3	5951.2(14)	7564(3)	6233.3(8)	41.2(5)
C1	10344(2)	15345(5)	6527.7(18)	108.0(14)
C2	9475(2)	15538(3)	6743.8(13)	76.0(9)
C44	8857.0(18)	14122(3)	6623.7(10)	58.5(7)
C4	9109(2)	12755(3)	6413.9(13)	72.5(9)
C5	8542(2)	11474(3)	6329.1(12)	69.8(8)
C6	7696.6(17)	11544(3)	6449.9(9)	50.7(6)
C7	7053.5(17)	9221(3)	6775.2(9)	51.2(6)
C8	6426.7(15)	7875(3)	6695.4(8)	44.5(5)
C9	6323.2(17)	6907(3)	7075.3(9)	52.3(6)
C10	5756.5(17)	5592(3)	7001.6(9)	51.3(6)
C11	5294.3(15)	5226(3)	6556.8(9)	45.2(5)
C12	4715.1(15)	3798(3)	6498.2(9)	46.4(5)
C13	4862.6(17)	2577(3)	6182.9(10)	53.5(6)
C14	4325.4(17)	1249(3)	6120.6(10)	54.2(6)
C15	3607.0(16)	1175(3)	6377.5(9)	46.8(6)
C16	2804.3(16)	-1308(3)	6054.2(10)	50.7(6)
C17	2738.7(19)	-1149(3)	5564.4(10)	58.8(7)
C18	2536.3(19)	-2432(3)	5267.1(10)	61.4(7)
C19	2399.3(17)	-3914(3)	5448.7(10)	55.0(6)
C20	2128(2)	-5287(3)	5105.4(13)	74.0(8)
C21	2318(5)	-6818(5)	5339(2)	173(2)
C22	1178(2)	-5064(5)	4886.1(17)	111.8(15)
C23	4014.9(15)	3663(3)	6763.3(9)	45.4(5)
C24	3455.0(15)	2364(3)	6702.5(8)	43.9(5)
C25	2650.8(16)	1933(3)	6896.2(8)	46.3(5)
C26	2168.1(18)	2634(3)	7224.1(10)	57.9(7)
C27	1386.4(19)	1930(4)	7309.9(11)	66.3(7)
C28	1069.6(19)	574(4)	7063.7(11)	68.1(8)
C29	1538.7(18)	-153(3)	6744.9(10)	60.5(7)
C30	2340.8(16)	525(3)	6668.0(9)	49.2(6)
C31	5372.2(15)	6231(3)	6158.0(8)	43.7(5)
C32	4873.1(16)	6032(3)	5692.0(9)	51.6(6)
C33	4957.8(18)	7046(3)	5321.6(10)	58.1(7)
C34	5552.2(17)	8304(3)	5393.0(9)	52.4(6)
C35	6039.5(16)	8572(3)	5842.0(9)	45.3(5)
C36	6620.2(18)	9971(3)	5916.2(9)	54.2(6)

Atom	*x*	*y*	*z*	U(eq)
C37	9016(3)	17034(4)	6531.6(17)	104.2(13)
C38	9705(4)	15660(5)	7294.7(16)	126.7(17)
C39	7994.7(19)	14150(3)	6741.0(11)	62.4(7)
C40	7422.5(18)	12878(3)	6656.2(10)	58.5(7)
C41	2698(2)	-2772(3)	6248.9(10)	62.5(7)
C42	2492(2)	-4058(3)	5945.9(11)	66.0(8)
C43	2676(3)	-5235(6)	4678.3(18)	126.8(15)

Supplementary Table 7. Hydrogen atom coordinates (Å ×10^4) and isotropic displacement parameters (Å^2 ×10^3) for CzNI.

Atom	*x*	*y*	*z*	U(eq)
H1A	10204	15252	6183	162
H1B	10652	14415	6657	162
H1C	10719	16249	6608	162
H4	9678	12693	6327	87
H5	8734	10565	6190	84
H9	6629	7122	7382	63
H10	5693	4950	7263	62
H13	5338	2661	6009	64
H14	4440	438	5915	65
H17	2831	-170	5431	71
H18	2490	-2299	4935	74
H21A	1882	-7032	5547	259
H21B	2904	-6801	5526	259
H21C	2290	-7624	5097	259
H22A	1004	-5890	4657	168
H22B	1112	-4063	4725	168
H22C	805	-5094	5135	168
H23	3921	4447	6982	54
H26	2369	3559	7382	69
H27	1066	2369	7536	80
H28	526	150	7117	82
H29	1327	-1070	6585	73
H32	4477	5191	5636	62
H33	4616	6892	5020	70
H34	5621	8966	5137	63
H37A	8866	16921	6188	156
H37B	9414	17913	6603	156
H37C	8481	17209	6670	156
H38A	10143	16473	7377	190
H38B	9941	14672	7423	190
H38C	9174	15911	7429	190
H39	7797	15053	6881	75

Atom	*x*	*y*	*z*	U(eq)
H40	6851	12931	6740	70
H41	2763	-2899	6582	75
H42	2414	-5040	6081	79
H43A	2531	-6137	4475	190
H43B	3304	-5246	4802	190
H43C	2533	-4291	4494	190

Supplementary Table 8. Fractional atomic coordinates ($\times 10^4$) and equivalent isotropic displacement parameters ($Å^2 \times 10^3$) for TPANI. U_{eq} is defined as 1/3 of the trace of the orthogonalised U_{IJ} tensor.

Atom	*x*	*y*	*z*	U(eq)
O2	2328.6(4)	2043.9(8)	1636.2(9)	35.0(3)
O1	2323.0(5)	4848.5(8)	2382.9(9)	41.4(4)
N2	2304.0(5)	3441.0(9)	2033.7(10)	26.2(3)
N1	4298.6(5)	3477.6(12)	9767.0(11)	39.1(4)
C24	2837.1(5)	3118.2(11)	4009.0(12)	23.0(4)
C29	2699.2(6)	2437.0(11)	3324.8(12)	23.9(4)
C22	2723.8(6)	3961.4(11)	3695.7(12)	24.3(4)
C25	3087.7(6)	2951.2(11)	5021.8(12)	25.0(4)
C19	3234.1(6)	3655.5(11)	5691.5(12)	26.6(4)
C16	3503.0(6)	3556.9(12)	6751.2(13)	27.9(4)
C21	2859.9(6)	4624.2(11)	4352.8(13)	28.0(4)
C28	2799.1(6)	1613.2(12)	3634.3(13)	29.5(4)
C26	3164.4(6)	2087.6(11)	5308.9(13)	28.6(4)
C23	2441.4(6)	4136.2(12)	2676.9(13)	26.9(4)
C30	2435.4(6)	2595.4(11)	2274.3(13)	26.6(4)
C31	2001.9(6)	3625.0(11)	1056.2(12)	26.7(4)
C27	3029.0(6)	1441.4(12)	4635.3(13)	30.6(4)
C20	3118.8(6)	4467.5(12)	5337.9(13)	30.0(4)
C15	3889.2(6)	3039.1(12)	7029.4(13)	32.5(5)
C34	1410.8(6)	4071.1(11)	-791.0(13)	29.9(4)
C7	4778.6(6)	3391.0(13)	9936.6(13)	32.7(5)
C35	1866.4(6)	3896.0(12)	-716.4(13)	33.0(5)
C14	4147.9(6)	3008.5(13)	8012.2(13)	34.7(5)
C36	2161.3(6)	3677.6(12)	193.5(13)	31.7(4)
C6	4098.6(6)	3565.3(12)	10601.1(13)	31.2(4)
C17	3392.4(6)	4043.7(12)	7512.0(13)	31.0(4)
C1	3676.3(6)	3214.1(12)	10574.5(14)	32.7(5)
C13	4030.6(6)	3501.4(13)	8760.3(13)	33.2(5)
C18	3649.5(6)	4022.6(13)	8491.4(13)	33.9(5)
C2	3486.7(7)	3299.4(12)	11396.5(14)	36.0(5)
C33	1259.7(6)	4006.8(13)	91.9(13)	37.3(5)
C32	1550.2(6)	3782.7(12)	1007.6(14)	35.6(5)
C37	1083.7(6)	4338.6(13)	-1790.6(14)	35.6(5)
C12	5023.3(7)	3934.2(13)	9474.6(15)	42.7(5)

C5	4325.2(7)	4002.6(13)	11457.6(14)	41.9(5)
C3	3710.6(7)	3740.1(14)	12243.4(15)	43.5(5)
C40	1277.1(7)	4136.6(15)	-2705.7(13)	45.2(5)
C4	4129.5(8)	4085.7(15)	12273.2(15)	49.8(6)
C11	5489.3(7)	3842.3(16)	9626.1(17)	52.3(6)
C8	5006.8(7)	2770.1(16)	10563.4(16)	53.3(6)
C10	5712.0(7)	3221.6(18)	10246.0(16)	56.3(7)
C39	631.4(7)	3862.3(16)	-1938.5(16)	55.2(7)
C38	1012.0(9)	5299.3(15)	-1757.1(17)	63.6(7)
C9	5472.4(8)	2692.6(19)	10715.1(17)	68.4(8)

Supplementary Table 9. Hydrogen atom coordinates (Å ×10^4) and isotropic displacement parameters (Å^2 ×10^3) for TPANI.

**Atom**	***x***	***y***	***z***	**U(eq)**
H21	2779	5190	4140	34
H28	2712	1160	3167	35
H26	3314	1955	5989	34
H27	3091	870	4848	37
H20	3218	4935	5777	36
H15	3975	2701	6529	39
H35	1980	3927	-1308	40
H14	4408	2650	8181	42
H36	2472	3564	223	38
H17	3132	4400	7348	37
H1	3517	2914	9991	39
H18	3566	4367	8990	41
H2	3199	3051	11376	43
H33	949	4120	67	45
H32	1438	3738	1599	43
H12	4870	4375	9049	51
H5	4615	4246	11486	50
H3	3577	3804	12802	52
H40A	1558	4462	-2658	68
H40B	1053	4291	-3329	68
H40C	1344	3528	-2714	68
H4	4287	4385	12859	60
H11	5654	4214	9296	63
H8	4845	2393	10893	64
H10	6032	3157	10351	68
H39A	689	3249	-1900	83
H39B	435	4003	-2603	83
H39C	481	4029	-1408	83
H38A	881	5439	-1186	95
H38B	804	5484	-2389	95
H38C	1305	5590	-1675	95
H9	5629	2263	11154	82

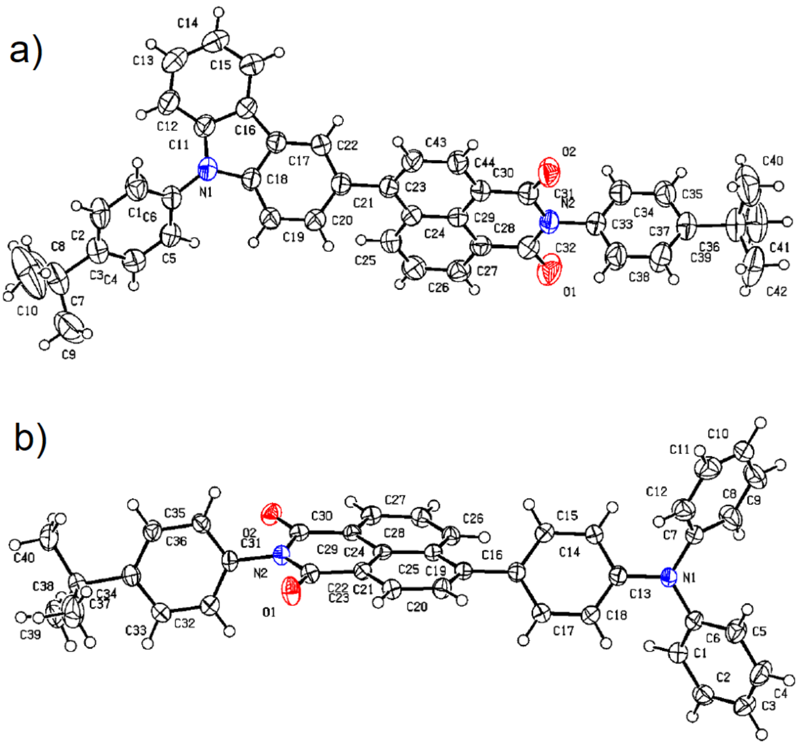

Supplementary Figure 12. ORTEP drawing. ORTEP drawing of a) compound CzNI and b) compound TPANI (ellipsoids at 50% probability).

The checkCIF results of compound indicates there is one A-level alert in its structure regarding solvent accessible voids. The solvent accessible voids (261 Å^3 per unit cells) can be attributed to the removal of solvent molecules before data collection because the boiling point of dichloromethane, the solvent used to cultivate single crystals, is low and easy to volatilize.

Supplementary Table 10. The NTO of TPANI in different excited states revealed by TDDFT calculations by employing the LC-*ω*PBE functional with the 6-31+G(d) basis set.

	Hole	Particle	Weight	Excited state transition nature	Excitation energy (eV)
S_1	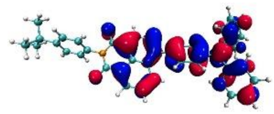	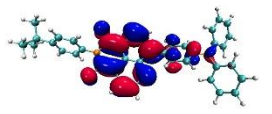	0.928	CT + ππ* (LE_A)	2.765
S_2	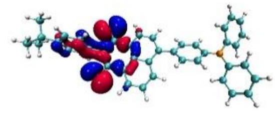	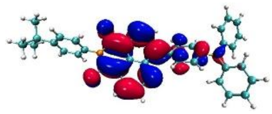	0.909	**nπ***+ ππ* (LE_A)	3.454
S_3	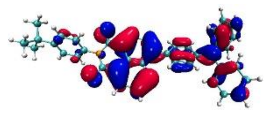	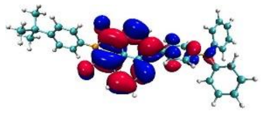	0.739	CT + ππ* (LE_A)	3.825
T_1	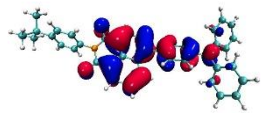	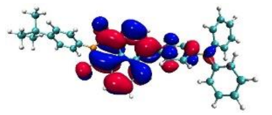	0.946	ππ* (LE_A)	1.727
T_2	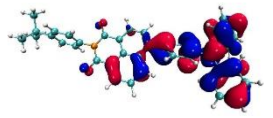	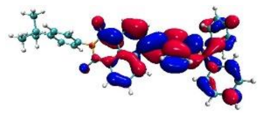	0.687	ππ* (LE_D) + ππ* (LE_A)	2.767
T_3	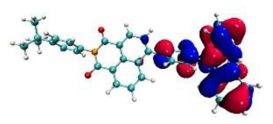	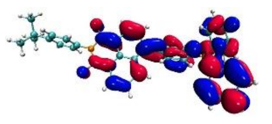	0.727	CT + ππ* (LE_D)	3.154
T_4	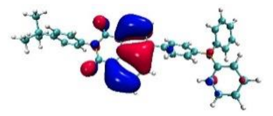	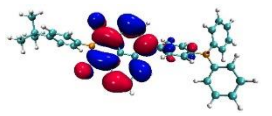	0.729	**ππ*** (LE_A)	3.352
T_5	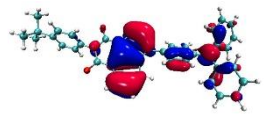	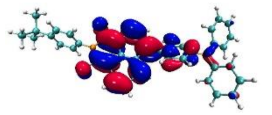	0.648	ππ* (LE_A) + CT	3.420
T_6	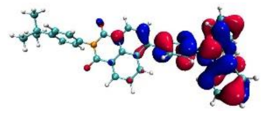	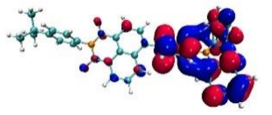	0.865	ππ* (LE_D)	3.608
T_7	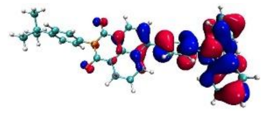	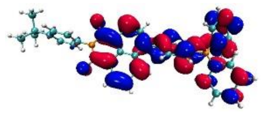	0.656	ππ* (LE_D) + ππ* (LE_A)	3.682
T_8	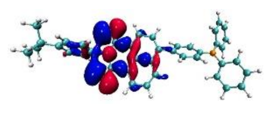	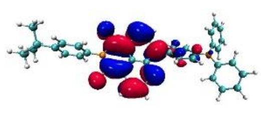	0.757	ππ* (LE_A) + nπ*	3.759

For S_2 excited state in TPANI, the "hole" is mainly located at carbonyl six-membered ring, while the "particle" is distributed on the naphthalene skeleton. Therefore, we can conclude that the $S_0 \rightarrow S_2$ transition is an nπ*-dominated transition nature. While for T_4 excited state in TPANI, both the "hole" and "particle" are mainly located at carbonyl six-membered ring and

naphthalene skeleton in TPANI. Therefore, we can conclude that the $S_0 \rightarrow T_4$ transition is a $\pi\pi^*$ transition nature.

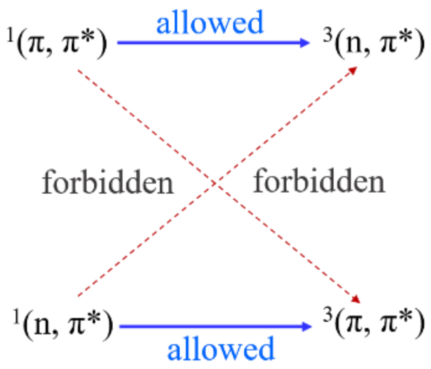

Supplementary Figure 13. El-Sayed's rule. Schematic diagram of El-Sayed's rule.

According to El-Sayed rule, the rate of RISC is also determined by the molecular orbital type of singlet and triplet states, for instance, a $\pi\pi^*$ triplet state could transition to an nπ^* singlet state, but not to a $\pi\pi^*$ triplet state, and an nπ^* triplet state could transition to a $\pi\pi^*$ triplet state, but not to an nπ^* triplet state and vice versa[4]. The NTO of $T_4 \rightarrow S_2$ in TPANI contains both the $\pi\pi^*$ and nπ^* transition character, which obeys El-sayed's rule and promotes the *m*-hRISC process from T_4 state to S_2 state. Meanwhile, the small ΔE_{ST} between T_4 and S_2 in TPANI also prompts the *m*-hRISC process.

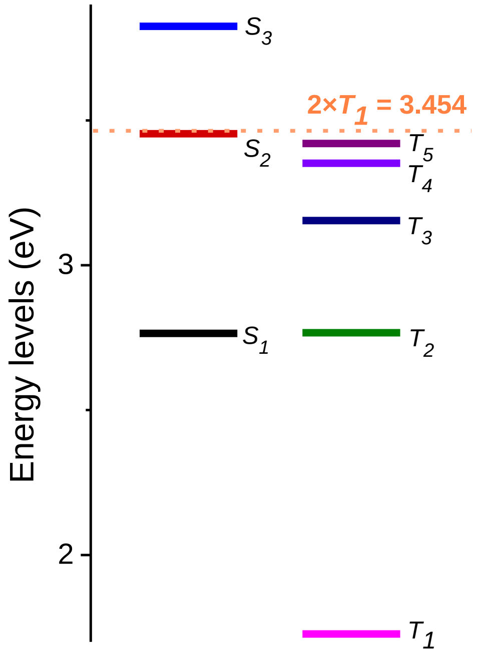

Supplementary Figure 14. Calculated excited state energy levels for TPANI. Simulated singlet and triplet state energy levels revealed by TD-DFT calculations by employing the LC-ωPBE functional with the 6-31+G(d) basis set.

Since the $T_4 \rightarrow S_2$ process is calculated to show an ultra-large *m*-hRISC rate of 2.1×10^9 s^{-1}, most of the T_4 excitons produced by triplet-channel TTA are estimated to be quickly converted to S_2 excitons. In view of the different transition characters of the T_4 (^3LE_A-dominated) and T_3 (^3LE_D-dominated) states of TPANI (vide Supplementary Table 10), the $T_4 \rightarrow T_3$ internal conversion (IC) process may be not quite fast. Although the T_2 state also displays some ^3LE_A-character, the relatively large $\Delta E_{(T_2T_4)}$ (~0.6 eV) should be adverse to the $T_4 \rightarrow T_2$ IC process. Consequently, both the $T_4 \rightarrow T_3$ and the $T_4 \rightarrow T_2$ IC processes may be not quite effective. Nevertheless, considering that the $T_2 \rightarrow S_1$ direct hRISC (*d*-hRISC) process has a moderate rate of 4.9×10^7 s^{-1} (vide Supplementary Table 11), once T_2 excitons are generated through direct electro-injection, the $T_2 \rightarrow S_1$ *d*-hRISC process could occur.

Supplementary Table 11. Calculated energy gap, spin–orbit coupling matrix element (SOCME) values between the singlet and triplet states, and the corresponding higher-lying reverse intersystem crossing rate constants (k_{hRISC}) of TPANI under Marcus reorganization energy of 0.20 eV at 300 K.

hRISC process	ΔES_mT_n (eV)	SOCME (cm^{-1})	k_{hRISC} (s^{-1})
$T_2 \rightarrow S_1$	-0.002^a	0.75	4.9×10^7
$T_3 \rightarrow S_2$	0.30	4.07	5.0×10^4
$T_4 \rightarrow S_2$	0.102	9.66	2.1×10^9
$T_5 \rightarrow S_2$	0.034	1.99	1.6×10^8

[a] Negative values indicate that the singlet energy level is lower than the triplet energy level.

The large SOC matrix element (SOCME) value of 9.66 cm^{-1} between T_4 and S_2 states of TPANI could be attributed to the quite different transition nature of T_4 ($\pi\pi^*$) and S_2 ($n\pi^*$) states.

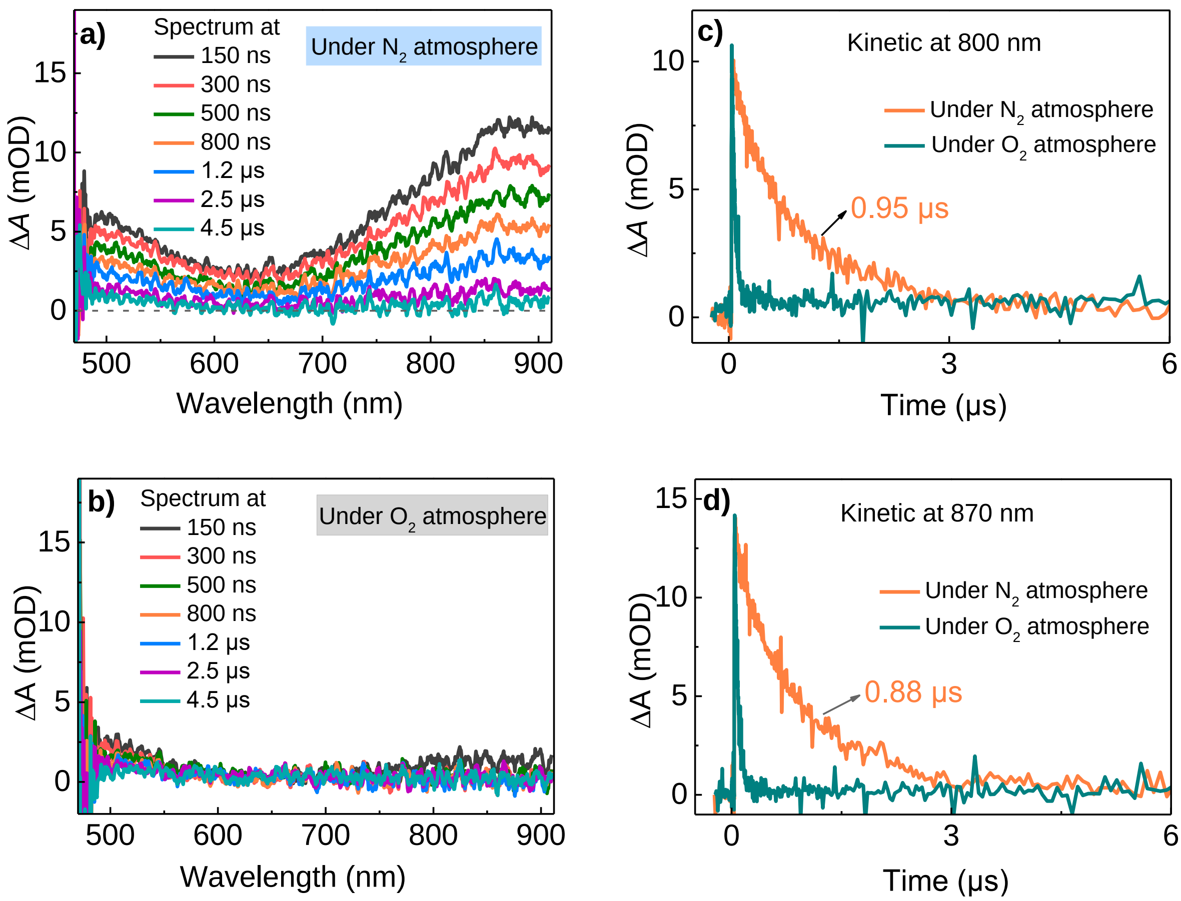

Supplementary Figure 15. TA spectra of TPANI. Transient absorption spectra a) under N_2 and b) under O_2 atmosphere. TA decay profiles at c) 800 nm and d) 870 nm of TPANI in iodomethane solutions (5×10^{-4} M) under N_2 and O_2 atmosphere at RT (λ_{ex} = 355 nm).

As illustrated in Supplementary Figure 15, in the absence of oxygen, TPANI manifests obvious excited state absorption (ESA) bands, and the TA decay profiles demonstrate the presence of significant long-lived excited species with lifetime of microsecond scales. However, when the atmosphere was changed from N_2 to O_2, the corresponding excited state absorption intensity is significantly decreased, indicating that both the two ESA bands can be attributed to the absorption of triplet states.

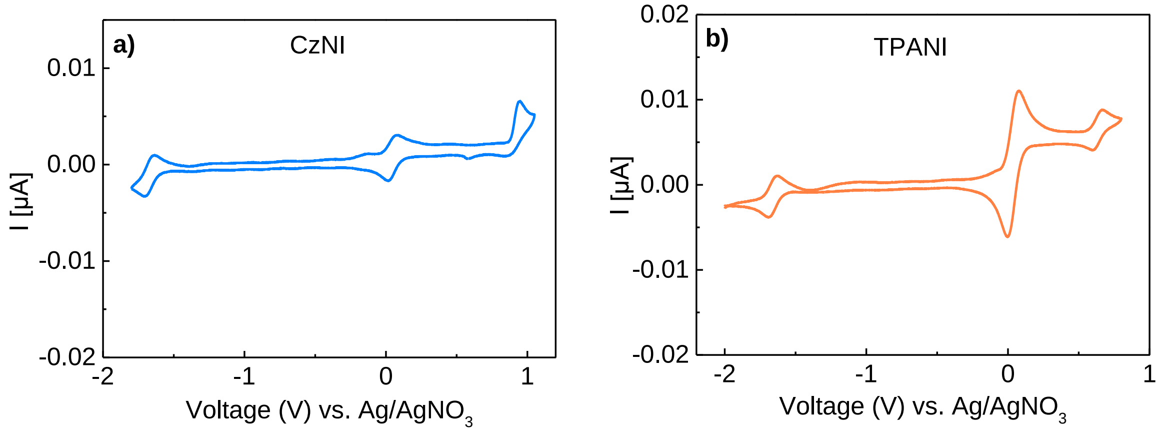

Supplementary Figure 16. CV spectra. Cyclic voltammogram of a) CzNI and b) TPANI in MeCN solution (5 × 10^{-4} M).

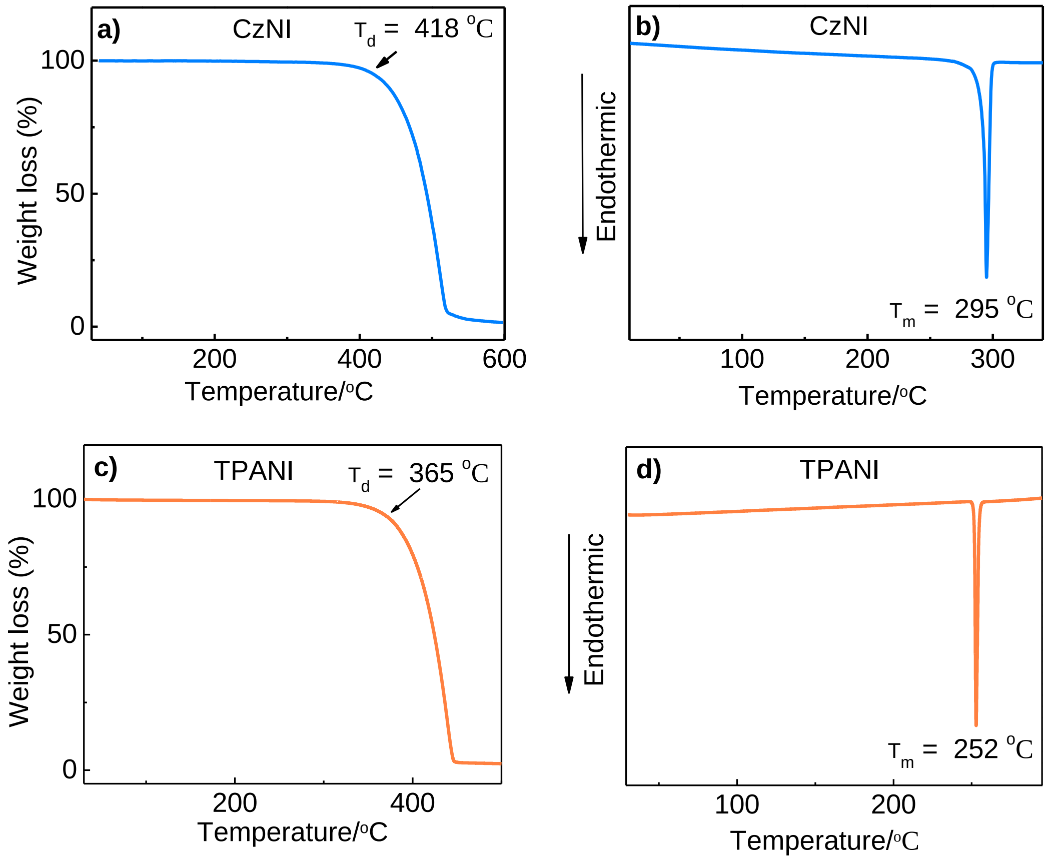

Supplementary Figure 17. Thermal stability of CzNI and TPANI. TGA thermogram of a) CzNI and c) TPANI; DSC thermograms of b) CzNI and d) TPANI.

Supplementary Note 3. Electroluminescence characterization

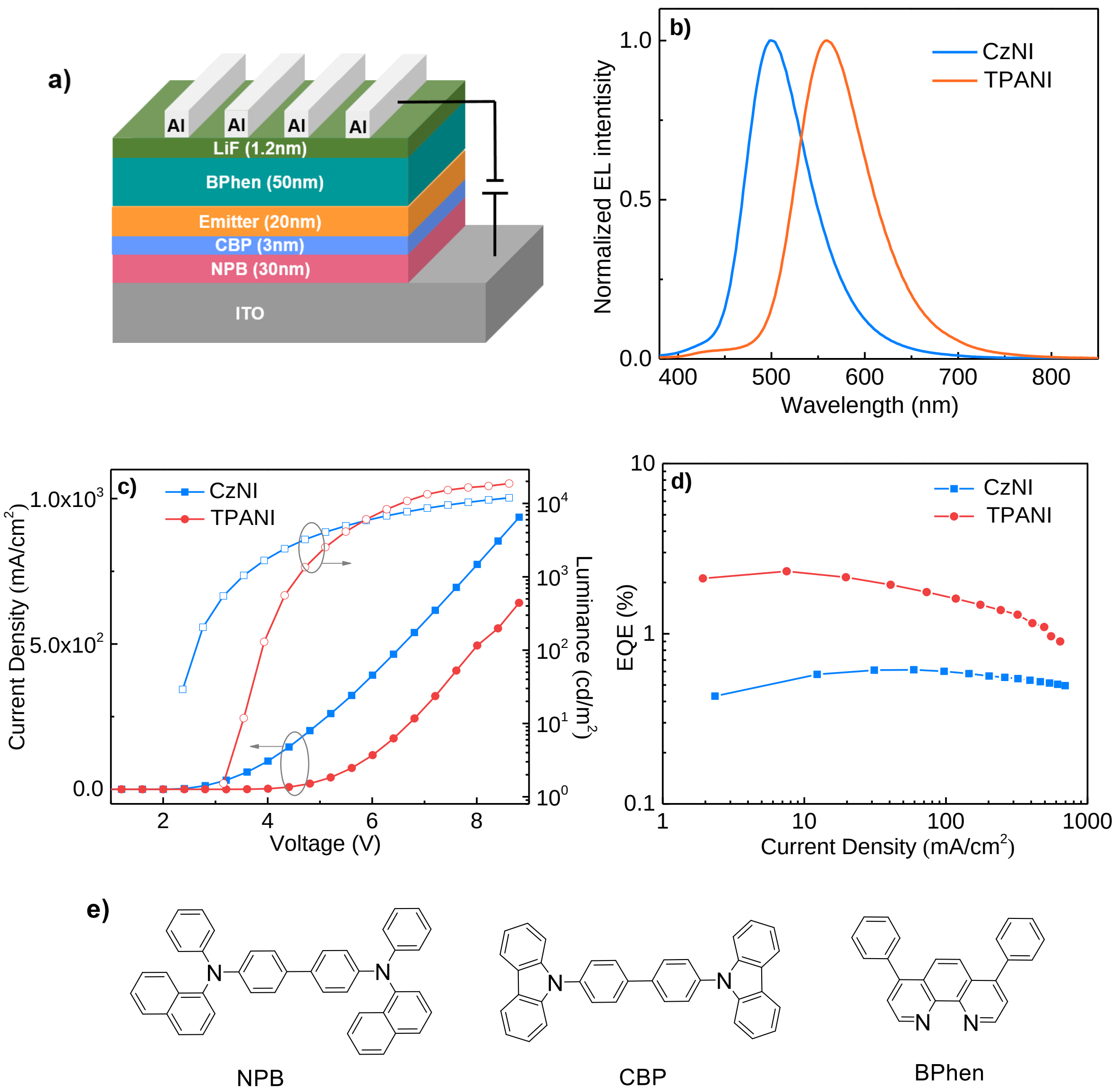

Supplementary Figure 18. EL characteristics of Device A and B. a) Device structure, b) EL spectra at current density of 641 and 1016 mA cm^{-2}, respectively, c) *J*-*V*-*L* characteristics, d) EQE-*J* curves of the CzNI and TPANI-based OLEDs, and e) chemical structures of the materials employed in the devices. Device structure: ITO/NPB (30 nm) /CBP (3 nm) /EML (20 nm) /BPhen (50 nm) /LiF (1.2 nm) /Al (120 nm), in which CzNI and TPANI were used as EML for Device A and B, respectively.

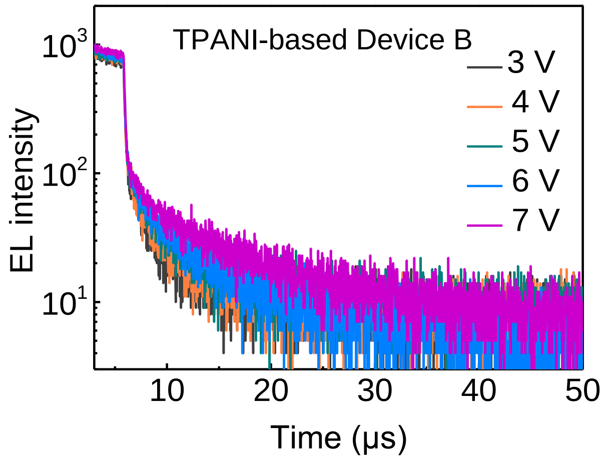

Supplementary Figure 19. Transient EL spectra at different driving voltages for TPANI-based Device B (pulse width: 5 μs).

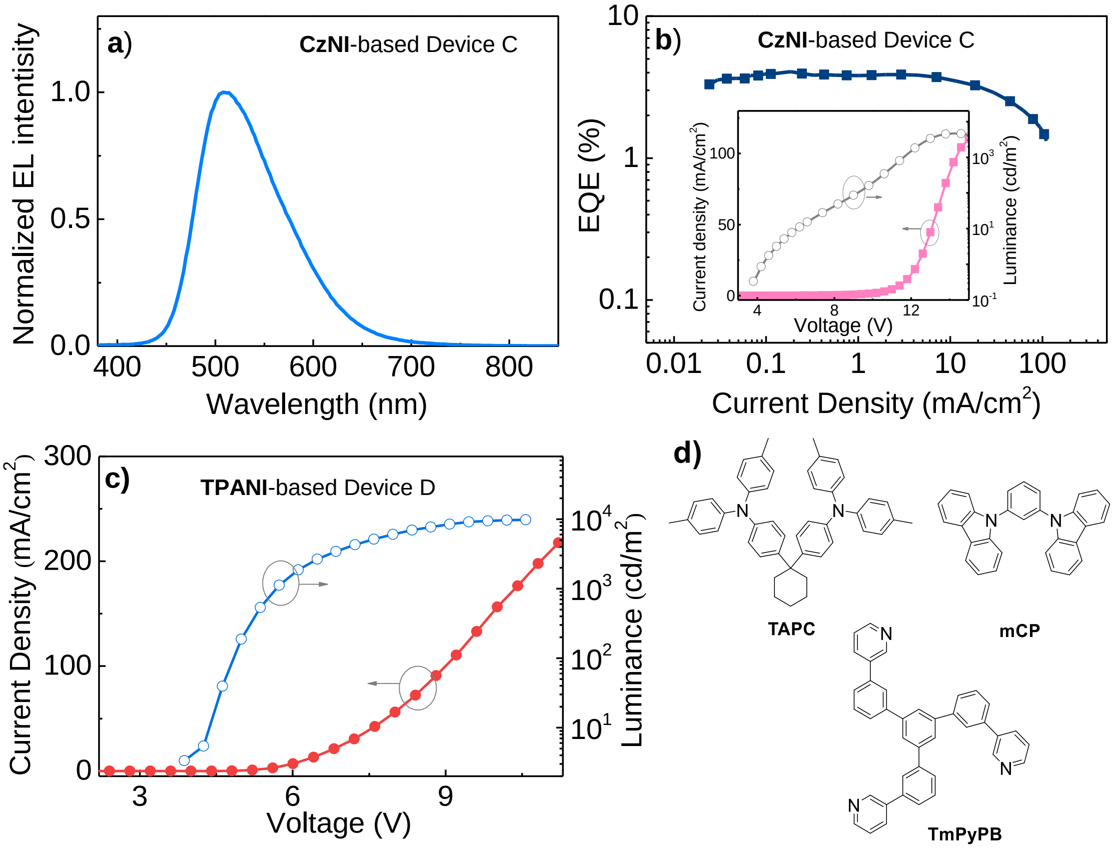

Supplementary Figure 20. EL characteristics of Device C and D. a) EL spectrum at current density of 110 mA cm^{-2}, b) EQE-*J* curves of the CzNI-based Device C (Inset: *J*-*V*-*L* characteristics). c) *J*-*V*-*L* characteristics of the TPANI-based Device D. d) Chemical structures of the materials employed in the Device C and D. Device structure: ITO/PEDOT:PSS (40 nm)/TAPC (20 nm)/mCP (10 nm) /EML (20 nm)/TmPyPB (50 nm)/LiF (1.2 nm)/Al (120 nm), in which CzNI and TPANI were used as EML for Device C and D, respectively.

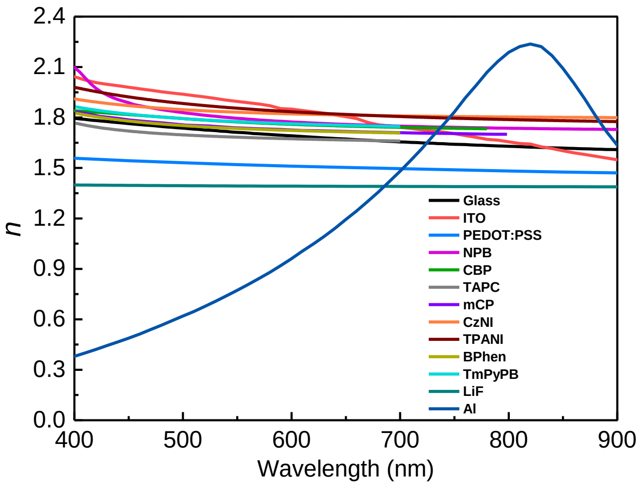

Supplementary Figure 21. *n*–*λ* spectra. Refractive indexes (*n*) at different wavelength of the materials used in the optical simulation.

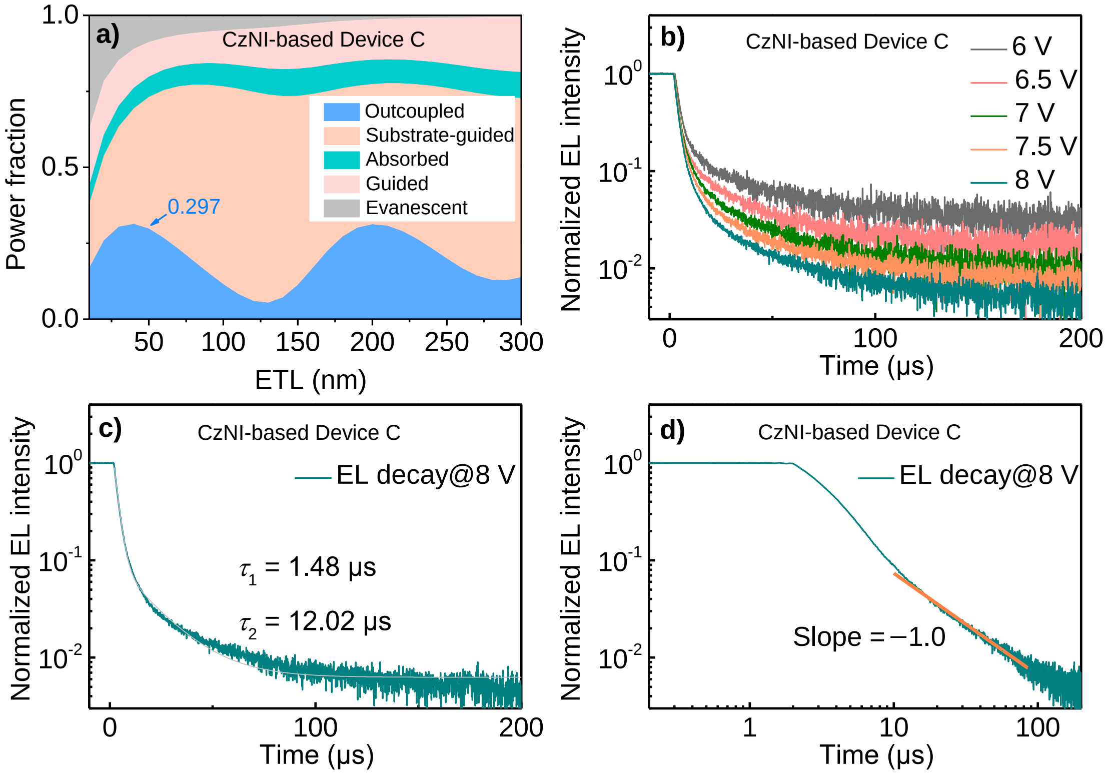

Supplementary Figure 22. Optical simulation and transient EL profiles of CzNI-based Device C. a) Simulated photon distributions of all loss channels as a function of the electron transport layer (ETL) thickness for CzNI-based Device C. The loss channels include substrate-guided mode, absorbed mode, guided mode and evanescent mode. b) Transient EL spectra at different driving voltages, c) the fitting result of the single-logarithmic EL decay curve, and d) the fitting result of the double-logarithmic EL decay profile in a time range of 10-90 μs of CzNI-based Deivce C (pulse width: 500 μs, bias: 8 V, a reverse-pulse voltage of −10 V was applied just after forward-pulse bias during the measurements).

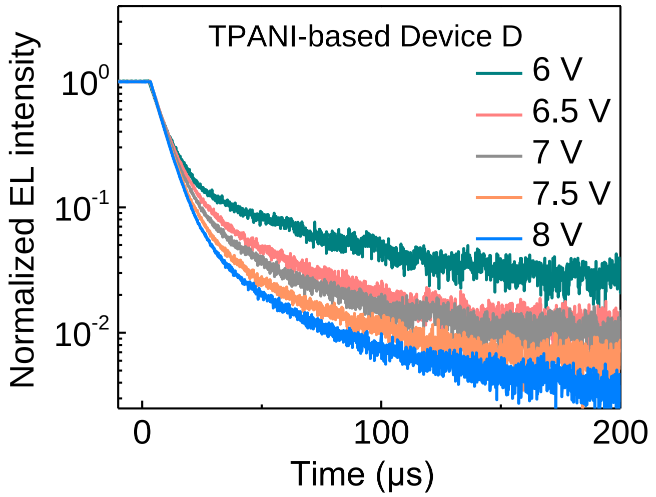

Supplementary Figure 23. Transient EL profiles of TPANI-based Device D. Transient EL spectra at different driving voltages for TPANI-based Device D (pulse width: 500 μs, a reverse-pulse voltage of −10 V was applied just after forward-pulse bias during the measurements).

In an OLED, EQE = $\gamma_{e\text{-}h} \times \varphi_{PL} \times EUE \times \eta_{out}$, where $\gamma_{e\text{-}h}$ is the the electron-hole balance ratio; φ_{PL} is the PL quantum efficiency of the emitting layer; EUE is the exciton utilization efficiency (namely total radiative singlet ratio); and η_{out} is the light out-coupling efficiency. Here, the η_{out} values of CzNI-based Device C and TPANI-based Device D are 29.7% and 33.1%, respectively (as shown in Figure 6 and Supplementary Figure 20), and the PLQY values of CzNI and TPANI neat film under N_2 atmosphere are 61.9% and 50.6%, respectively. Therefore, assuming the utilization of the maximum $\gamma_{e\text{-}h}$ value as 100%, the EUE_{max} of CzNI-based Device C and TPANI-based Device D are calculated to be 22.1% and 46.7%, respectively. Note that this EUE_{max} value of TPANI-based device is higher than the spin statistical limit of TTA-OLED whose singlet and triplet TTA channels are both opened (40.0%, vide Figure 1b), it can be deduced that additional TTA-m-hRISC process and/or direct hRISC (*d*-hRISC) process (from T_2 to S_1) should contribute to the triplet harvesting in this device.

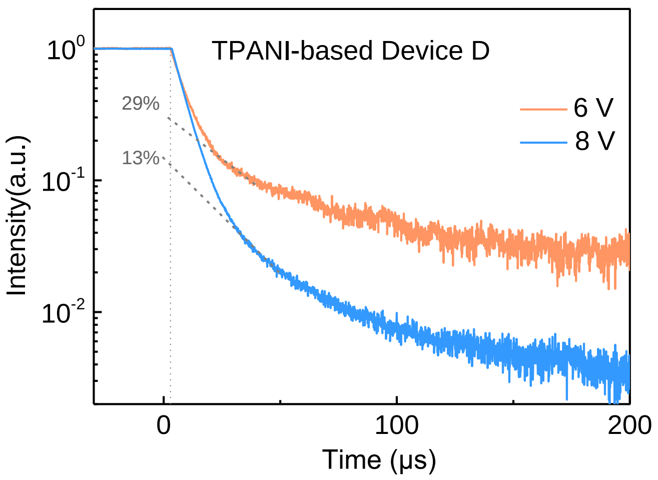

Supplementary Figure 24. Transient EL profiles. Transient EL spectra of TPANI-based Device D at 6 V and 8 V.

As shown in **Supplementary Figure 24**, the ratio of delayed-to-steady state emission (I_{delay}/I_{steady}) for TPANI-based Device D at 6 V is obviously larger than that at 8 V because the triplet excitons at higher driving voltage may be quenched by charges or go through other quenching processes which would lead to the decrease of delayed component.[5] Ultilizing the I_{delay}/I_{steady} value for Device D at 6 V, the proportion of singlet excitons *via* singlet-channeled TTA and triplet-channeled TTA-*m*-hRISC (η_{DF} is the proportion of singlet excitons generated *via* TTA-involved processes, *i.e.*, $\eta_{TTA} + \eta_{TTA\text{-}m\text{-}hRISC}$) could be calculated. If there is no direct hRISC (*d*-hRISC) process contributing to EL, according to the formula $I_{delay}/I_{steady} = \eta_{DF}/(\eta_S + \eta_{DF})$,[6, 7] where η_S is electrically generated singlet exciton (25.0%), the η_{DF} in Device D was calculated to be ca. 10.2%. Based on the fact that the sum of η_S and η_{DF} (35.2%) is much lower than the EUE_{max} (46.7%) of Device D, it can be deduced that the direct hRISC (*d*-hRISC) process should also contribute to the triplet utilization in this device. In this case, according to the updated formula $I_{delay}/I_{steady} = \eta_{DF}/(\eta_S + \eta_{DF} + \eta_{d\text{-}hRISC})$, the η_{DF} in Device D could be re-calculated to be ca. 13.5% (29% × 46.7%), and thus the corresponding singlet exciton generation proportion from *d*-hRISC process ($\eta_{d\text{-}hRISC}$) was calculated to be 8.2%.

Supplementary Note 4. Equations

Supplementary equations for the TTA model

When TTA process occurs, after pulse off immediately, the T_1 density can be expressed as follows[8]

$$\frac{\mathrm{d}[T(t)]}{\mathrm{d}t} = -k_T\,[T(t)] - \gamma_{TT}\,[T(t)]^2 \tag{1}$$

where k_T is the monomolecular decay rate constant of the triplet excitons, $T(t)$ is the T_1 density at time of t, γ_{TT} is the rate constant of bimolecular TTA process of T_1 excitons. At high triplet densities, $\gamma_{TT}[T(t)]^2 >> k_T[T(t)]$, thus Supplementary Eequation 1 can be expressed as follows

$$\frac{\mathrm{d}[T(t)]}{\mathrm{d}t} = -\gamma_{TT}\,[T(t)]^2 \tag{2}$$

Supplementary Equation 2 can be further expressed as below:

$$\int \frac{1}{[T(t)]^2} \cdot \mathrm{d}[T(t)] = \int -\gamma_{TT}\,\mathrm{d}t \tag{3}$$

the solution of Supplementary Equation 3 is given by Supplementary Equation 4

$$\frac{1}{[T(t)]} = \gamma_{TT}\,t + \mathrm{C} \tag{4}$$

C is a constant which equals $\frac{1}{[T(0)]}$,thus Supplementary Equation 4 can be expressed as below:

$$\frac{1}{[T(t)]} = \gamma_{TT}\,t + \frac{1}{[T(0)]} \tag{5}$$

The intensity of the delayed fluorescence (I_{DF}) induced by the TTA-involved processes can be expressed as Supplementary Equation 6:

$$I_{\mathrm{DF}} \propto [T(t)]^2 = \left(\gamma_{TT}\,t + \frac{1}{[T(0)]}\right)^{-2} \tag{6}$$

As shown in Figure 4i and 6f, the EL decay profile of TPANI-based devices in double-log form was well-fitted by the above TTA model, verifying that triplet excitons can be harvested through TTA-involved processes in this device. In the case of CzNI-based Device C, however, only a slope of −1.0 could be observed in the similar time region (vide Supplementary Figure 22d), excluding the significant TTA-involved triplet exciton utilization in this device.

Supplementary equations for triplet dynamics process

The states in Figure 7 can be expressed as follows:

$$\frac{\mathrm{d}[S_1]}{\mathrm{d}t} = \frac{1}{4}G + 0.082G - (k_r + k_{nr})[S_1] + k_{IC}[S_m] \quad (7)$$

$$\frac{\mathrm{d}[T_1]}{\mathrm{d}t} = \frac{3}{4}G - 0.082G - 2\frac{1}{4}k_1[T_1]^2 - 2\frac{3}{4}k_1[T_1]^2 - k_{nr}^{T}[T_1] + k_{IC}^{T_2}[T_n] + 2k_{-1}[^1(TT)] + 2k_{-1}[^3(TT)] \quad (8)$$

$$\frac{\mathrm{d}[^1(TT)]}{\mathrm{d}t} = \frac{1}{4}k_1[T_1]^2 - k_{-1}[^1(TT)] - k_S[^1(TT)] \quad (9)$$

$$\frac{\mathrm{d}[^3(TT)]}{\mathrm{d}t} = \frac{3}{4}k_1[T_1]^2 - k_{-1}[^3(TT)] - k_T[^3(TT)] \quad (10)$$

$$\frac{\mathrm{d}[T_n]}{\mathrm{d}t} = k_T[^3(TT)] - k_{m\text{-hRISC}}[T_n] - k_{IC}^{T_n}[T_n] \quad (11)$$

$$\frac{\mathrm{d}[S_m]}{\mathrm{d}t} = k_S[^1(TT)] + k_{m\text{-hRISC}}[T_n] - k_{IC}[S_m] \quad (12)$$

where $[S_1]$, $[T_1]$, $[T_n]$, $[S_m]$, $[^1(TT)]$, $[^3(TT)]$ are the densities of the S_1, T_1, T_n, S_m excitons, and singlet-featured and triplet-featured intermediate states in sequence; G is the term for exciton generation; k_1 and k_{-1} are the rate constants of the generation of TT pair *via* the collision of two T_1 excitons and the dissociation of TT pair respectively; k_S and k_T are the rate constants of internal conversion (IC) process from ^{1}(TT) and ^{3}(TT) intermediate states to S_m and T_n respectively; $k_{m\text{-hRISC}}$ is the rate constant of hRISC process from T_n to S_m state in the TTA-*m*-hRISC process; k_{IC} and $k_{IC}^{T_n}$ are the rate constants of IC processes from S_m to S_1 and from T_n to T_2 state, respectively; k_{nr} and k_{nr}^{T} are the rate constants of non-radiative processes from S_1 to S_0 and from T_1 to S_0, respectively.

For the steady state condition,

$$\frac{\mathrm{d}[{}^1(\mathrm{TT})]}{\mathrm{d}t} = \frac{\mathrm{d}[{}^3(\mathrm{TT})]}{\mathrm{d}t} = \frac{\mathrm{d}[\mathrm{S}_m]}{\mathrm{d}t} = \frac{\mathrm{d}[\mathrm{T}_n]}{\mathrm{d}t} = \frac{\mathrm{d}[\mathrm{S}_1]}{\mathrm{d}t} = \frac{\mathrm{d}[\mathrm{T}_1]}{\mathrm{d}t} = 0 \quad (13)$$

Thus,

$$[{}^1(\mathrm{TT})] = \frac{1}{4}\frac{k_1}{k_{-1}+k_\mathrm{S}}[\mathrm{T}_1]^2 \quad (14)$$

$$[{}^3(\mathrm{TT})] = \frac{3}{4}\frac{k_1}{k_{-1}+k_\mathrm{T}}[\mathrm{T}_1]^2 \quad (15)$$

$$[\mathrm{T}_n] = \frac{k_\mathrm{T}}{k_\mathrm{IC}^{\mathrm{T}_n}+k_{m\text{-hRISC}}}[{}^3(\mathrm{TT})] = \frac{3}{4}\frac{k_\mathrm{T}}{k_\mathrm{IC}^{\mathrm{T}_n}+k_{m\text{-hRISC}}}\frac{k_1}{k_{-1}+k_\mathrm{T}}[\mathrm{T}_1]^2 \quad (16)$$

$$[\mathrm{S}_m] = \frac{k_\mathrm{S}}{k_\mathrm{IC}}[{}^1(\mathrm{TT})] + \frac{k_{m\text{-hRISC}}}{k_\mathrm{IC}}[\mathrm{T}_n] = \frac{1}{4}\frac{k_\mathrm{S}}{k_\mathrm{IC}}\frac{k_1}{k_{-1}+k_\mathrm{S}}[\mathrm{T}_1]^2 + \frac{3}{4}\frac{k_{m\text{-hRISC}}}{k_\mathrm{IC}}\frac{k_\mathrm{T}}{k_\mathrm{IC}^{\mathrm{T}_n}+k_{m\text{-hRISC}}}\frac{k_1}{k_{-1}+k_\mathrm{T}}[\mathrm{T}_1]^2 \quad (17)$$

Thus, the ratio of the $\eta_{\mathrm{TTA}\text{-}m\text{-hRISC}}$ (the proportion of singlet excitons *via* triplet-channel TTA process, namely TTA-*m*-hRISC) to η_TTA (the proportion of singlet excitons *via* singlet-channel TTA process) can be expressed as follows:

$$\frac{\eta_{\mathrm{TTA}\text{-}m\text{-hRISC}}}{\eta_\mathrm{TTA}} = \frac{k_{m\text{-hRISC}}[\mathrm{T}_n]}{k_\mathrm{S}[{}^1(\mathrm{TT})]} = \frac{\frac{3}{4}k_{m\text{-hRISC}}\frac{k_\mathrm{T}}{k_\mathrm{IC}^{\mathrm{T}_n}+k_{m\text{-hRISC}}}\frac{k_1}{k_{-1}+k_\mathrm{T}}[\mathrm{T}_1]^2}{\frac{1}{4}k_\mathrm{S}\frac{k_1}{k_{-1}+k_\mathrm{S}}[\mathrm{T}_1]^2}$$

$$= \frac{\frac{3}{4}k_{m\text{-hRISC}}\frac{k_\mathrm{T}}{k_\mathrm{IC}^{\mathrm{T}_n}+k_{m\text{-hRISC}}}\frac{1}{k_{-1}+k_\mathrm{T}}}{\frac{1}{4}k_\mathrm{S}\frac{1}{k_{-1}+k_\mathrm{S}}} \quad (18)$$

The k_{-1} can be negligible in amorphous film due to the very low diffusion rate of triplet excitons.[8] Thus, Supplementary Equation 18 can be further expressed as below:

$$\frac{\eta_{\mathrm{TTA}\text{-}m\text{-hRISC}}}{\eta_\mathrm{TTA}} = \frac{3\,k_{m\text{-hRISC}}}{k_\mathrm{IC}^{\mathrm{T}_n}+k_{m\text{-hRISC}}} \quad (19)$$

Considering that the $k_{m\text{-hRISC}}$ of TPANI is calculated to be as large as 2.1 × 10^9 s^{-1}, and meanwhile the relatively large calculated $\Delta E_{(\mathrm{T_2T_4})}$ (~0.6 eV) may lead to a relatively slow $T_4 \rightarrow T_2$ IC process, it is assumed that $k_{m\text{-hRISC}} >> k_\mathrm{IC}^{\mathrm{T}_n}$, and thus the $\eta_{\mathrm{TTA}\text{-}m\text{-hRISC}}/\eta_\mathrm{TTA}$ is 3. Consequently, the total η_DF of 13.5% in Device D can be divided into two parts: η_TTA of ca. 3.4% and $\eta_{\mathrm{TTA}\text{-}m\text{-hRISC}}$ of ca. 10.1%.

The $k_{m\text{-hRISC}}$ derived from second-order perturbation theory can be expressed as follows[8]

$$k_{m\text{-hRISC}} = \frac{2\pi}{\hbar} \left| \langle \Psi_{S_m+S_0} | \hat{H}_{SOC} | \Psi_{^3(TT)} \rangle + \sum_{n} \frac{\langle \Psi_{S_m+S_0} | \hat{H}_{SOC} | \Psi_{T_n+S_0} \rangle \langle \Psi_{T_n+S_0} | \hat{H}_{el} | \Psi_{^3(TT)} \rangle}{E_{^3(TT)} - E_{T_n+S_0}} \right|^2 \delta(E_{S_m+S_0} - E_{^3(TT)}) \quad (20)$$

where $\Psi_{S_m+S_0}$, $\Psi_{^3(TT)}$, and $\Psi_{T_n+S_0}$ are the wave functions of each state. Similarly, $E_{S_m+S_0}$, $E_{^3(TT)}$ are the energy levels of each state. $\hat{H}_{SOC}$ and $\hat{H}_{el}$ are the Hamiltonians for spin-orbit and electron-electron interactions, respectively. According to Supplementary Equation 20, the interactions between ^{3}(TT) and S_m can be mediated by T_n state. For the large $k_{m\text{-hRISC}}$, the energy gap between ^{3}(TT) and T_n states should have a small value, while the RISC process from T_n to S_m states should possess large spin–orbit coupling (SOC) interactions. For TPANI, TD-DFT calculations indicated that 2 × T_1 and T_4 states displayed a relatively small energy gap of 0.0102 eV, meanwhile T_4 and S_2 states showed large SOC matrix element (SOCME) value of 9.66 cm^{-1}. As a reslut, a large $k_{m\text{-hRISC}}$ of 2.1×10^9 s^{-1} could be realized.

Supplementary Note 5. ^{1}H, ^{13}C NMR spectra and High resolution mass spectrometry

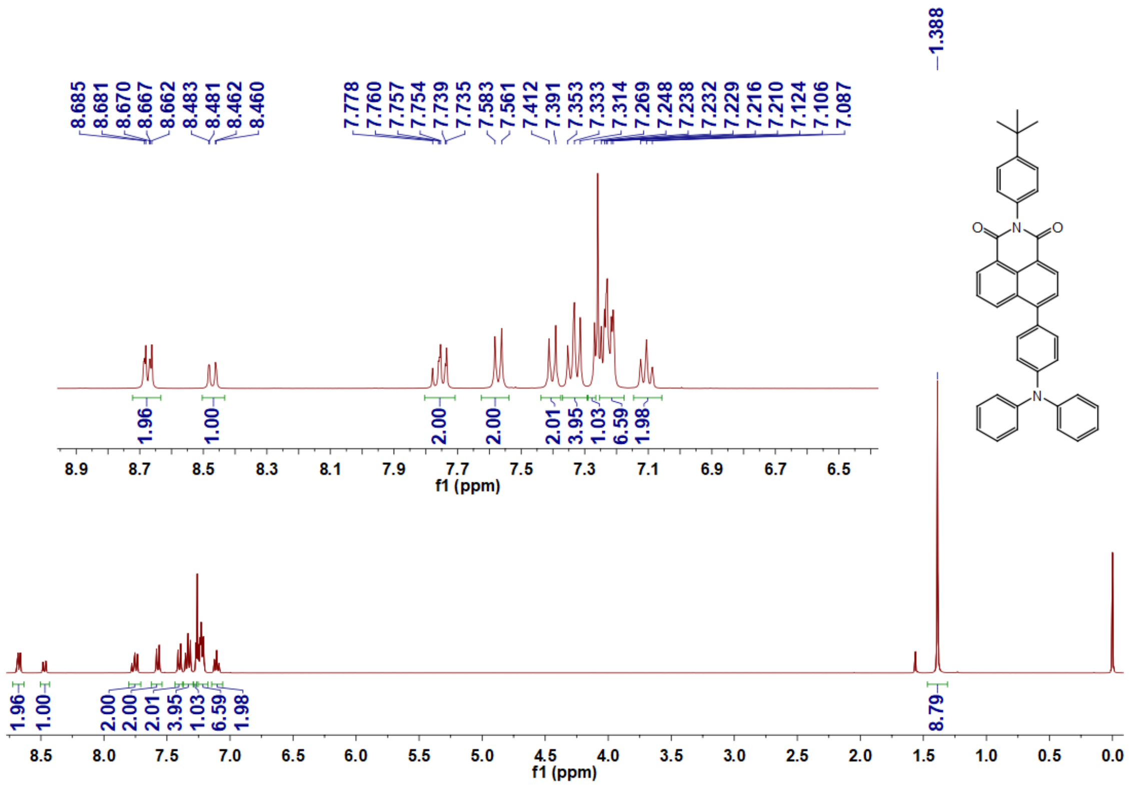

Supplementary Figure 25. The ^{1}H NMR spectrum of TPANI in $CDCl_3$.

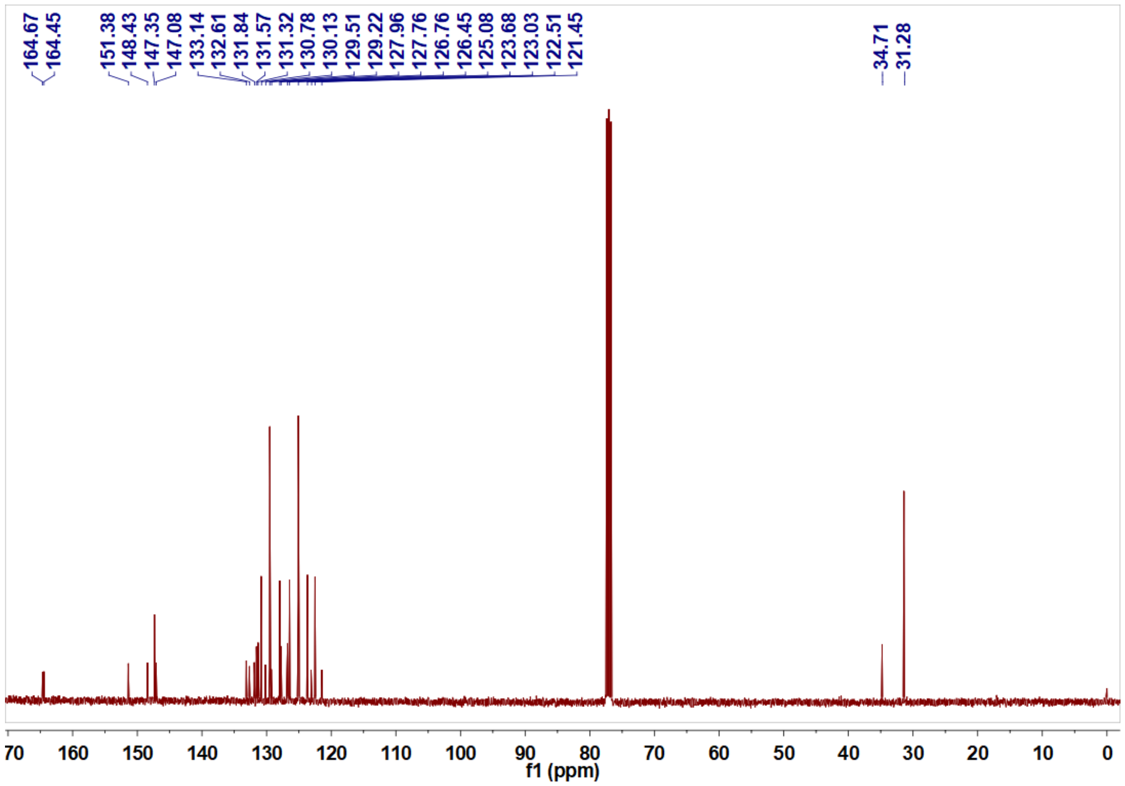

Supplementary Figure 26. The ^{13}C NMR spectrum of TPANI in $CDCl_3$.

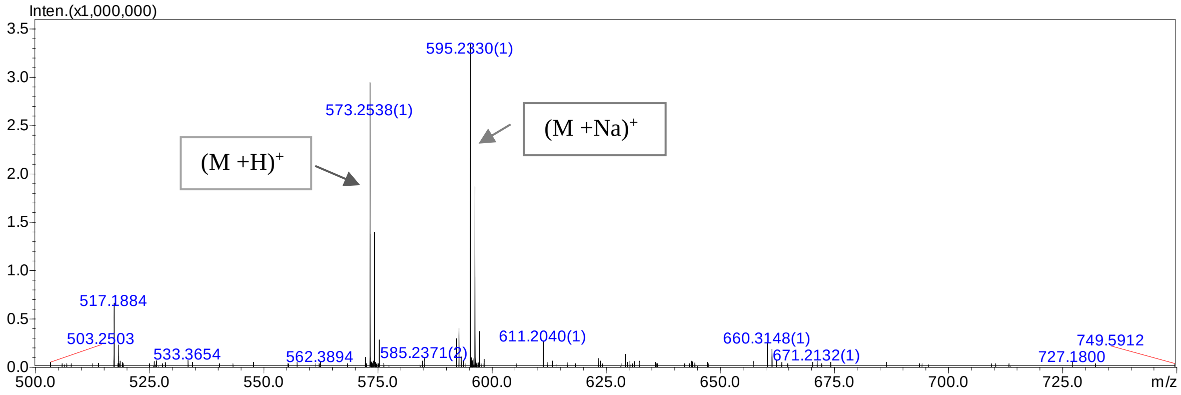

Supplementary Figure 27. The high resolution ESI mass spectrum of TPANI.

Supplementary References

1. Zhou, J. et al. Charge-transfer-featured materials-promising hosts for fabrication of efficient oleds through triplet harvesting via triplet fusion. *Chem. Commun.* **50**, 7586-7589 (2014).
2. Wang, Y. et al. An efficient guest/host fluorescent energy transfer pair based on the naphthalimide skeleton, and its application in heavily-doped red organic light-emitting diodes. *Dyes Pigm.* **100**, 87-96 (2014).
3. Zhang, H. et al. High-performance ultraviolet organic light-emitting diode enabled by high-lying reverse intersystem crossing. *Angew. Chem. Int. Ed.* **60**, 22241-22247 (2021).
4. Zhang, W. et al. Enhancing fluorescence of naphthalimide derivatives by suppressing the intersystem crossing. *J. Phys. Chem. C* **121**, 23218-23223 (2017).
5. Tang, X. et al. Efficient nondoped blue fluorescent organic light-emitting diodes (OLEDs) with a high external quantum efficiency of 9.4% @ 1000 cd m^{-2} based on phenanthroimidazole−anthracene derivative. *Adv. Funct. Mater.* **28**, 1705813 (2018).
6. Wang, Y. et al. Molecular engineering of anthracene-based emitters for highly efficient nondoped deep-blue fluorescent OLEDs. *J. Mater. Chem. C* **8**, 9678-9687 (2020).
7. Kang, S., Huh, J.-S., Kim, J.-J. & Park, J. Highly efficient deep-blue fluorescence OLEDs with excellent charge balance based on phenanthro[9,10-d]oxazole-anthracene derivatives. *J. Mater. Chem. C* **8**, 11168-11176 (2020).
8. Lim, H., Woo, S.J., Ha, Y.H., Kim, Y.H. & Kim, J.J. Breaking the efficiency limit of deep-blue fluorescent OLEDs based on anthracene derivatives. *Adv. Mater.* **34**, 2100161 (2022).
